# The disruptor of telomeric silencing 1-like (DOT1L) promotes peritoneal fibrosis through the upregulation and activation of protein tyrosine kinases

**DOI:** 10.1186/s43556-023-00161-z

**Published:** 2024-01-04

**Authors:** Min Tao, Yingfeng Shi, Hui Chen, Jinqing Li, Yi Wang, Xiaoyan Ma, Lin Du, Yishu Wang, Xinyu Yang, Yan Hu, Xun Zhou, Qin Zhong, Danying Yan, Andong Qiu, Shougang Zhuang, Na Liu

**Affiliations:** 1 Department of Nephrology, Pudong New District, Shanghai East Hospital, Tongji University School of Medicine, 150 Jimo Road, Shanghai, 200120 China; 2 Shanghai Key Laboratory of Maternal and Fetal Medicine, Clinical and Translational Research Center of Shanghai First Maternity & Infant Hospital, School of Life Sciences and Technology, Tongji University, Shanghai, China; 3 Department of Medicine, Rhode Island Hospital and Alpert Medical School, Brown University, Providence, RI USA

**Keywords:** Disruptor of telomeric silencing 1-like, Di-methylation of histone H3 on lysine-79, Epidermal growth factor receptor, Janus kinase 3, Peritoneal fibrosis

## Abstract

The disruptor of telomeric silencing 1-like (DOT1L), a specific histone methyltransferase that catalyzed methylation of histone H3 on lysine 79, was associated with the pathogenesis of many diseases, but its role in peritoneal fibrosis remained unexplored. Here, we examined the role of DOT1L in the expression and activation of protein tyrosine kinases and development of peritoneal fibrosis. We found that a significant rise of DOT1L expression in the fibrotic peritoneum tissues from long-term PD patients and mice. Inhibition of DOT1L significantly attenuated the profibrotic phenotypic differentiation of mesothelial cells and macrophages, and alleviated peritoneal fibrosis. Mechanistically, RNA sequencing and proteomic analysis indicated that DOT1L was mainly involved in the processes of protein tyrosine kinase binding and extracellular matrix structural constituent in the peritoneum. Chromatin immunoprecipitation (ChIP) showed that intranuclear DOT1L guided H3K79me2 to upregulate EGFR in mesothelial cells and JAK3 in macrophages. Immunoprecipitation and immunofluorescence showed that extranuclear DOT1L could interact with EGFR and JAK3, and maintain the activated signaling pathways. In summary, DOT1L promoted the expression and activation of tyrosine kinases (EGFR in mesothelial cells and JAK3 in macrophages), promoting cells differentiate into profibrotic phenotype and thus peritoneal fibrosis. We provide the novel mechanism of dialysis-related peritoneal fibrosis (PF) and the new targets for clinical drug development. DOT1L inhibitor had the PF therapeutic potential.

**Supplementary Information:**

The online version contains supplementary material available at 10.1186/s43556-023-00161-z.

## Introduction

Current estimates indicated that approximately 15% of end-stage renal disease (ESRD) patients were treated with peritoneal dialysis (PD) worldwide [1]. Depending on the intact peritoneum structure, patient outcomes with PD were comparable to or better than those with hemodialysis [2–4]. However, long-term exposure of peritoneum to the high glucose, toxicity or inflammation caused membrane dysfunction and fibrosis [5–7]. Nearly 50% of PD patients eventually developed progressive peritoneal fibrosis (PF) and ultrafiltration decline, which had no established treatment [1, 8].

Fibrosis was a hallmark and common pathway that contributed to the decline of peritoneal ultrafiltration [9]. Myofibroblast, a kind of activated fibroblasts characterized by expression of alpha-smooth muscle actin (a-SMA) and deposition of fibrillar collagen during tissue or organ fibrosis [10]. Myofibroblasts were heterogeneous and could originate from several sources, including proliferation of local resident fibroblasts, epithelial-mesenchymal transition (EMT), and a newly identified phenomenon macrophage-myofibroblast transition etc [10, 11].

Mesothelial cells (MCs), special type of epithelial cells, were considered as the main victims of the peritoneal injury during PD [6, 12]. In response to the stimuli of dialysate, peritoneal MCs progressively lost epithelial phenotype and acquired fibroblast-like characteristic [12]. MCs with profibrotic phenotype tended to proliferate and migrate more efficiently and expressed less adhesive and tight junction proteins, leading to MCs denudation and fibers replacement [13]. This process could be conducted by the interaction or transactivation of multiple growth factor receptor signaling pathways [14]. Our previous study demonstrated the vital role of epithelial growth factor receptor (EGFR) in the process of PF [15].

On the other hand, immune cells, particularly macrophages, were also easy to find in the fibrotic peritoneum [16]. The function of macrophages in peritoneal fibrosis (PF) is uncovering. Macrophage infiltrating at the early time point after an acute injury exhibited primarily a pro-inflammatory M1 phenotype, but switched to a anti-inflammatory and profibrotic M2 phenotype during the chronic insult, such as dialysis [17] Macrophages M2 polarization facilitated the fibrotic self-repair of the peritoneum [18, 19]. Interleukin (IL)-4 was considered as the main inducer for macrophage M2 differentiation [17]. IL-4 bound to its receptor complexes and activated Janus kinase (JAK), increasing the phosphorylation of signal transducer and activator of transcription 6 (STAT6) [20]. Increasing evidence indicated that macrophage M2 differentiation was mediated by histone lysine methyltransferases [21, 22].

The disruptor of telomeric silencing 1-like (DOT1L) was also a kind of lysine methyltransferase that catalyzes mono-, di- and trimethylation of histone H3 on lysine-79 (H3K79me1/me2/me3) in mammals [23–25]. It had been reported that DOT1L could maintain leukemic gene expression, promote the phenotype differentiation of neuronal, myocardial cells and breast cancer cells [26]. Inhibition of DOT1L activity could alleviate renal and pulmonary fibrosis [27, 28]. Moreover, DOT1L inhibitor, EPZ5676, had been evaluated for leukemia in clinical trial [29]. However, whether DOT1L is responsible for peritoneal fibrosis has not been elucidated. In current study, we suggested that DOT1L regulated the phenotype of mesothelial cells and macrophages through the upregulation and activation of protein tyrosine kinases. And DOT1L-based approaches were vital for investigating novel antifibrotic therapies, and improving clinical outcomes for PD patients.

## Results

### DOT1L is involved in the peritoneal fibrosis in patients and mice

**Fig. 1:**
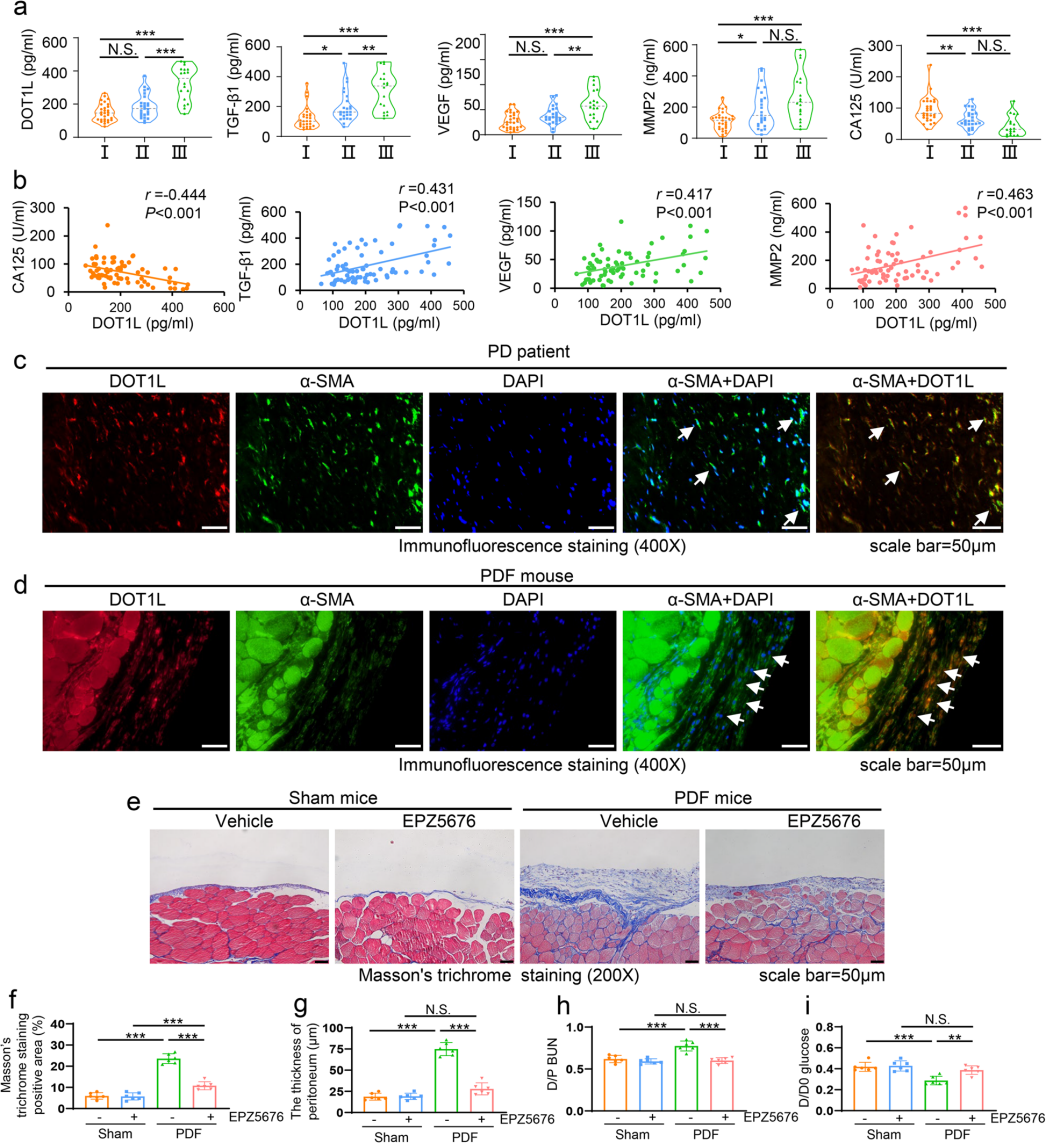
DOT1L is highly detected in dialysate effluent of long-term PD patients and mainly expressed in peritoneal cells with fibrotic phenotype, regulating the development of peritoneal fibrosis induced by PDF in mice. **a** Violin plot showed the expression levels of DOT1L, TGF-β1, VEGF, MMP2 and CA125 in peritoneal dialysis effluent according to ELISA. These patients were divided into three groups according to duration, Group I duration < 12 months (*n* = 29), Group II 12 months ≤ duration < 36 months (*n* = 25) and Group III 36 months ≤ duration (*n* = 18). **b** Correlations between DOT1L and levels of CA125, TGF-β1, VEGF and MMP2 in PD effluent. **c**, **d** Co-immunofluorescence photomicrographs illustrated co-staining of DOT1L and α-SMA in the peritoneum from patient and mouse with peritoneal fibrosis induced by PDF. Arrows indicated α-SMA-positive cells. **e** Photomicrographs showed Masson’s trichrome staining of the peritoneum in each group. **f** the bar graph showed the positive area of the Masson-positive submesothelial area (blue) from 10 random fields of peritoneal samples from six mice. **g** the bar graph showed the thickness of the compact zone measured from 10 random fields of peritoneal samples from six mice. **h** the D/P ratio of BUN. **i** the D/D0 ratio of glucose. Scale bar = 50μm. Data are means ± sem of 6 samples, *P < 0.05, **P < 0.01, *** *P* < 0.001, *P* ≥ 0.05 is not significant (NS)

We firstly investigated the expression level of DOT1L and its relationship with peritoneal injury factors in the dialysate effluent from PD patients (*n* = 72). As shown in Fig. 1a, enzyme-linked immunosorbent assay (ELISA) indicated that the longer duration on dialysis, the higher expression of DOT1L, transforming growth factor-β1 (TGF-β1), vascular endothelial growth factor (VEGF) and matrix metalloproteinase2 (MMP2), the lower expression of cancer antigen 125 (CA125), indicated the mesothelial cell mass [30]. Further analysis showed that the expression of DOT1L was positively related to the factors (TGF-β1, VEGF and MMP2), and negatively correlated with CA125 in PD effluent of patients (Fig. 1b). To investigate the source of DOT1L, we performed the immunofluorescent staining of DOT1L and α-SMA, the myofibroblast marker, in the thickened peritoneum of human and mice. As shown in Fig. 1c, d, DOT1L was abundantly expressed in the cytoplasm and nucleus of α-SMA-positive cells, a spindle or stellate shape, characterized by numerous cytoplasmic projections [10, 11]. To further elucidate the role of DOT1L in the development of PF, we set two mouse models of PF induced by 4.25% high glucose (HG) peritoneal dialysis fluid (PDF) or 0.1% chlorhexidine gluconate (CG). Masson trichrome staining showed repeated injection of PDF or CG resulted in the increased thickness of the submesothelial area, and interstitial expansion with fibers and collagens accumulation. Treatment with DOT1L specific inhibitor EPZ5676 significantly attenuated these pathological changes (Figs. 1e-g and S1a-c). In addition, we evaluated the functional alteration of peritoneal membrane by the ratio of urea nitrogen in dialysate-to-plasm (D/P) and the glucose absorption rate by the ratio of dialysate glucose at 2 h after PDF injection to dialysate glucose at 0 h (D/D0). DOT1L inhibitor effectively improved peritoneal function by reducing D/P ratio of urea nitrogen and improving D/D0 ratio of glucose (Figs. 1h, i and S1d, e). Collectively, these data suggested that DOT1L of dialysate was mainly from fibrotic phenotype cells of peritoneum and it played a vital role in the development of peritoneal fibrosis.

### DOT1L is involved in protein tyrosine kinase (PTK) binding and extracellular matrix protein (ECM) structural constituent in the peritoneum

**Fig. 2:**
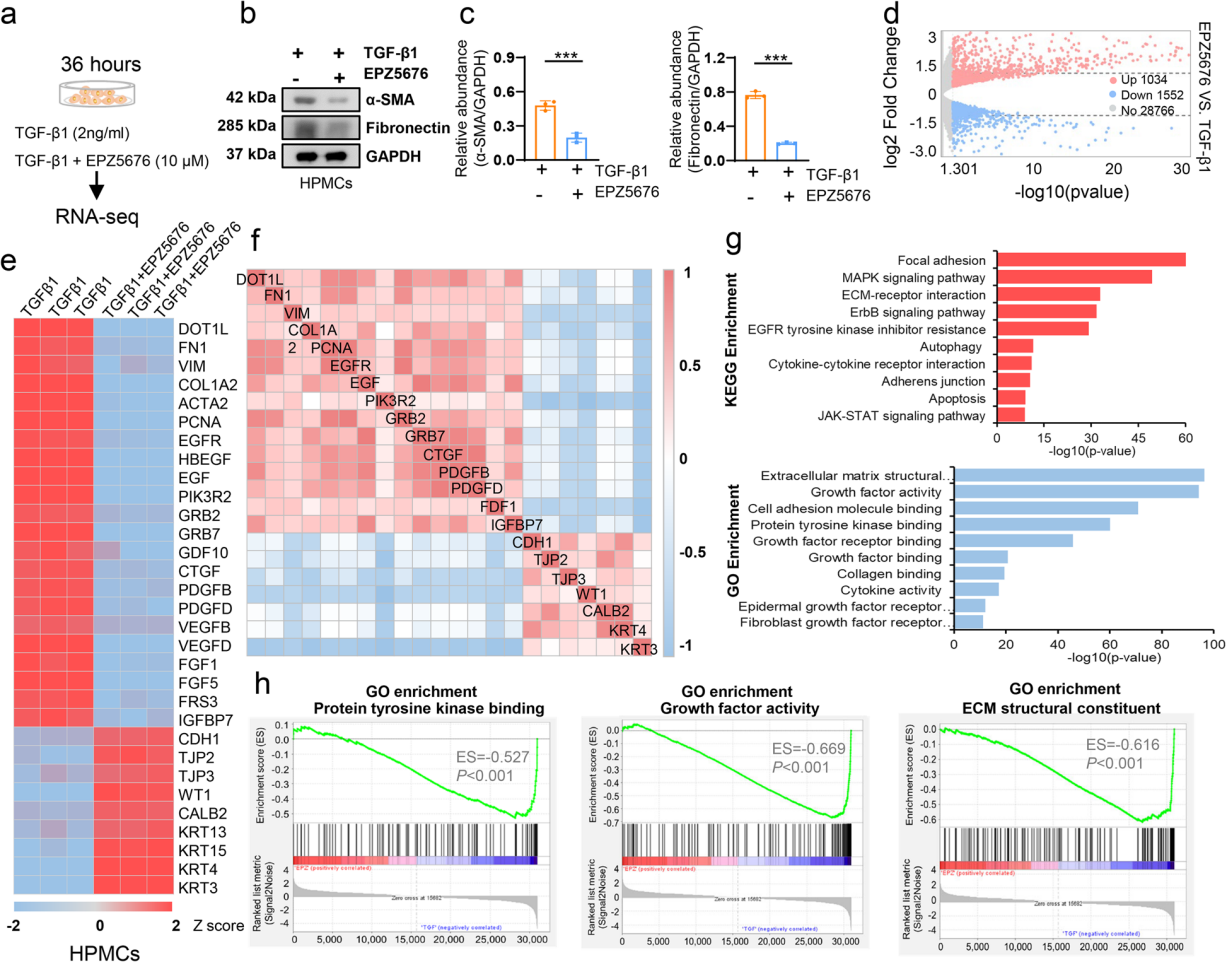
DOT1L is involved in process of PTK binding, growth factor activity and ECM structural constituent in peritoneal mesothelial cells. **a** 2 ng/ml TGF-β1-stimulated HPMCs with or without 10μM EPZ5676 treatment. **b** Cell lysates were subjected to immunoblotting analysis with antibodies against α-SMA, fibronectin and GAPDH. **c** Expression levels of α-SMA and fibronectin were quantified by densitometry and normalized with GAPDH. **d** Cell lysates were subjected to RNA-sequencing. Volcano plot of RNAs from TGF-β1-injured HPMCs treated with or without EPZ5676. The red or blue dots represented RNAs in HPMCs that are up-regulated or down-regulated, respectively. **e** Heat map showed differential expressed genes in each group with three repeats. Differential gene expression was displayed as Z-score. **f** Correlation heat map showed the correlation between differential expressed genes. Red represented positive correlation and blue represented negative correlation. **g** KEGG analysis for the genes with altered RNA expressions in the cluster bar graph. GO analysis for the genes with altered RNA expressions in the cluster bar graph. **h** GSEA plots for the gene enrichment with altered RNA expressions based on GO database

Mesothelial cells had been considered as the main victims of long-term PD [6, 12]. Evidence indicated that mesothelial cell after trans-differentiation was the important source of cells with profibrotic phenotype [31]. TGF-β1 was a well-established fibrotic driver for multiple organs fibrosis [32], and we used it to set the in vitro model of human peritoneal mesothelial cells (HPMCs) (Fig. 2a-c). To identify the targeted genes regulated by DOT1L in the HPMCs, RNA-seq was analyzed in 2ng/ml TGF-β1-stimulated HPMCs with or without EPZ5676 treatment. Results showed that 1034 mRNAs revealed increased abundance and 1552 mRNAs exhibited decreased abundance owing to the DOT1L inhibitor (Fig. 2d). Compared to the TGF-β1 group, the mRNAs of FN1, COL1A2, ACTA2, and EGFR in the EPZ5676 treatment group exhibited an approximately 2 Z-score decrease (Fig. 2e). The RNA level of DOT1L was positively correlated with FN1, VIM, COL1A2 and EGFR, while negatively correlated with CDH1, TJP2, TJP3, etc. (Fig. 2f). Kyoto encyclopedia of genes and genomes (KEGG) analysis showed that significantly changed genes were involved in regulating focal adhesion, mitogen-activated protein kinase (MAPK) signaling pathway, ECM-receptor interaction and ErbB signaling, etc. (*P* < 0.001). Gene ontology (GO) analysis indicated that significantly changed genes were involved in regulating the ECM structural constituent, growth factor activity, cell adhesion molecule binding, PTK binding, etc. (*P* < 0.001) (Fig. 2g). Gene set enrichment analysis (GSEA) based on GO database showed that these differentially expressed genes were mainly involved in the PTK binding, growth factor activity, ECM structural constituent, etc. (*P* < 0.001) (Fig. 2h).

**Fig. 3:**
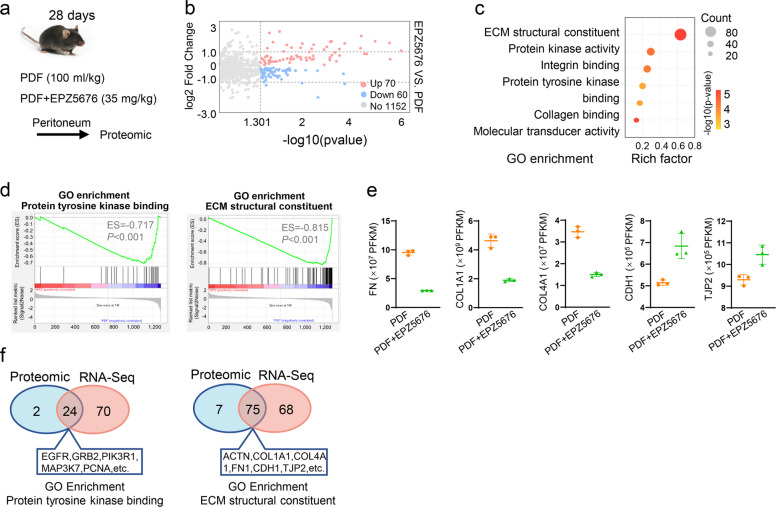
DOT1L is involved in process of PTK binding and ECM structural constituent in peritoneal tissues. **a** Proteomic analysis was carried out in the peritoneum from PDF-injured mice with or without EPZ5676 treatment. **b** Volcano plot of peritoneal protein from PDF-injured mice treated with or without EPZ5676. The red or blue dots represented proteins in peritoneum that are up-regulated or down-regulated, respectively. **c** Bubble graph showed the enrichment of genes with altered protein expressions based on GO database. **d** GSEA plots for the gene enrichment with altered protein expressions based on GO database. **e** Dot plots showed the expression of FN, COL1A1, CLO4A1, CDH1 and TJP2. **f** Venn plots showed the number of genes with altered protein and RNA in two enrichments. There were 3 samples in each group

To further verify these potential targets mediated by DOT1L in the peritoneal fibrosis, proteomic analysis was carried out in PDF-injured peritoneum from the mice with or without EPZ5676 treatment (Fig. 3a). There were statistical 70 proteins revealed increased protein abundance and 60 proteins exhibited decreased protein abundance owing to the DOT1L inhibitor (Fig. 3b). Bubble diagram and GSEA based on GO database showed that these differentially expressed proteins were mainly involved in the PTK binding and ECM structural constituent (Fig. 3c, d). Compared to the peritoneum under PDF injury, DOT1L inhibitor significantly reduced the protein levels of FN, COL1A1, COL4A1 and improved the protein of CDH1 and TJP2 (Fig. 3e). In the pathway of PTK binding, 24 genes revealed the difference both in RNA and protein levels, including EGFR (Fig. 3f). In the pathway of ECM structural constituent, 75 genes showed RNA and protein difference (Fig. 3f). Based on these data, it could be inferred that DOT1L potentially functions as a histone methyltransferase which promoted the mesothelial cell to acquire fibroblast-like phenotype through proceeding gene transcription of PTKs.

### DOT1L regulates the expression and activation of EGFR in mesothelial cells

**Fig. 4:**
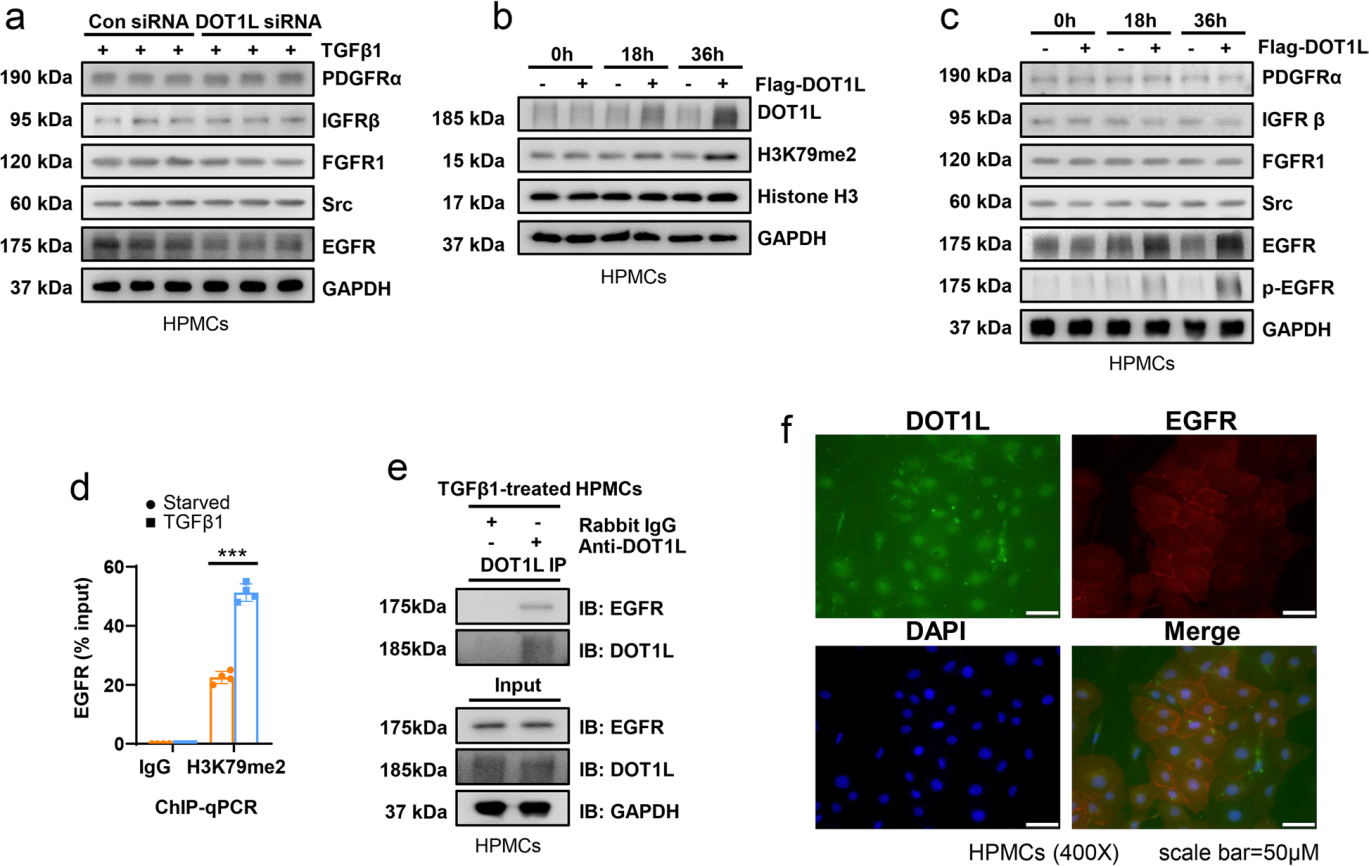
DOT1L regulates the expression and activation of EGFR in peritoneal mesothelial cells. **a** HPMCs were transfected with scrambled siRNA and DOT1L siRNA for 6 h, and then incubated with 2ng/ml TGF-β1 for an additional 36 h before being harvested for analysis. Cell lysates were subjected to immunoblotting analysis with antibodies against PDGFRα, IGFRβ, FGFR1, Src, EGFR and GAPDH. **b** HPMCs were transfected with Flag-DOT1L pcDNA3.0 plasmid or vector for 0, 18, 36 h and then subjected to immunoblotting analysis with antibodies against DOT1L, H3K79me2, Histone H3, and GAPDH **c** HPMCs were transfected with Flag-DOT1L pcDNA3.0 plasmid or vector for 0, 18, 36 h and then subjected to immunoblotting analysis with antibodies against PDGFRα, IGFRβ, FGFR1, Src, EGFR, p-EGFR and GAPDH. **d** ChIP-qPCR analysis of H3K79me2 enrichment in the EGFR promoter region. **e** 2ng/ml TGF-β1-treated HPMCs were subjected to immunoprecipitation with IgG or DOT1L antibody, followed by EGFR and DOT1L immunoblotting. Input lysates were analyzed by EGFR, DOT1L and Histone H3 immunoblotting. **f** Immunofluorescence co-staining of DOT1L and EGFR in TGF-β1-treated HPMCs. Scale bar = 50μm. Data are means ± sem of 3 samples, *P < 0.05, **P < 0.01, *** *P* < 0.001, *P* ≥ 0.05 is not significant (NS)

H3K79 was the histone substrate of DOT1L, which could be catalyzed into H3K79me1/me2/me3 in mammals [23]. Among those modification, H3K79me2 was an active marker associated with the gene transcription [33]. As indicated in RNA-seq and proteomic analysis above, we found that DOT1L regulated the transcription of PTKs, which included EGFR, platelet derived growth factor receptor (PDGFR), insulin like growth factor receptor (IGFR), fibroblast growth factor receptor (FGFR) and Src etc. [34]. To find the specific gene that transcription and translation were regulated by DOT1L-mediated H3K79me2, we examined the expression of a variety of PTKs in 2ng/ml TGF-β1-treated HPMCs with or without DOT1L siRNA. Immunoblotting results showed that DOT1L siRNA decreased EGFR expression by 50% in TGF-β1-stimulated HPMCs compared with them treated with scramble siRNA. There was no change for PDGFα, IGFRβ, FGFR1 and Src in the absent or present of DOT1L (Fig. 4a). In line with these observations, overexpression of DOT1L increased the protein levels of DOT1L and EGFR after 18 h of DOT1L intervene, and they reached the peak at 36 h (Fig. 4b,c). Chromatin immunoprecipitation (ChIP) quantitative polymerase chain reaction (qPCR) analysis further demonstrated that DOT1L regulated EGFR expression by H3K79me2 in TGF-β1-treated HPMCs (Fig. 4d). Notably, DOT1L overexpression not only increased the total EGFR, but also promoted its activation, marked by increased phosphorylation level (Fig. 4c). We speculated that DOT1L might interact with EGFR and affect its activity. To verify the hypothesis, we employed an cell model, exposing HPMCs to TGF-β1 and a DOT1L antibody, aiming to identify its interacting protein. Immunoblot analysis revealed the presence of EGFR in the DOT1L immunoprecipitated complexes, whereas it was absent in the negative control IgG complexes (Fig. 4e). The dual immunofluorescence staining showed that DOT1L is expressed in EGFR positive cells (Fig. 4f). These data suggested that DOT1L not only regulated the EGFR expression but also improved EGFR activation.

**Fig. 5:**
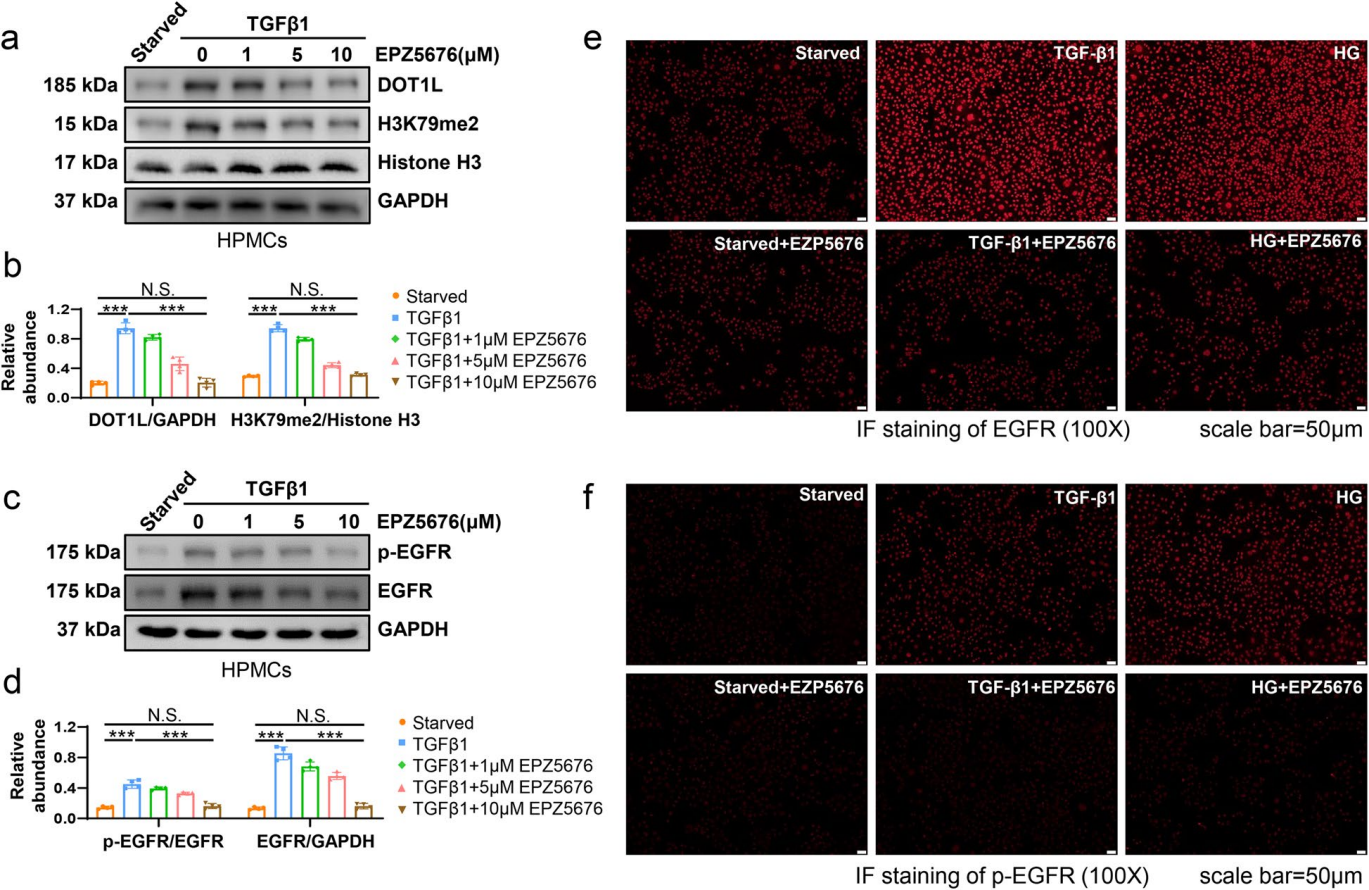
DOT1L inhibitor EPZ5676 reduces the expression and activation of EGFR in peritoneal mesothelial cells. **a** Serum-starved HPMCs were cultured in 2ng/ml TGF-β1 for 36 h with different concentrations of EPZ5676 (0, 1, 5, 10 μM). Cell lysates were subjected to immunoblotting analysis with specific antibodies against DOT1L, H3K79me2, Histone H3 or GAPDH. **b** Expression levels of DOT1L and H3K79me2 were quantified by densitometry and normalized with GAPDH, Histone H3, respectively. **c** Cell lysates were subjected to immunoblotting analysis with specific antibodies against p-EGFR, EGFR or GAPDH. **d** Expression levels of p-EGFR and EGFR were quantified by densitometry and normalized with EGFR and GAPDH, respectively. **e** Immunofluorescence of EGFR in HPMCs under different treatments (starved, 2ng/ml TGF-β1 or 60mM HG) with or without 10μM EPZ5676. **f** Immunofluorescence of p-EGFR in HPMCs under different treatments with or without 10μM EPZ5676. Scale bar = 50μm. Data are means ± sem of 4 samples, *P < 0.05, **P < 0.01, *** *P* < 0.001, *P* ≥ 0.05 is not significant (NS)

To further verify this results, in vitro HPMCs were injured by 2ng/ml TGF-β1 and treated by DOT1L inhibitor EPZ5676 at different doses (1, 5, 10 μM). Immunoblotting indicated that TGF-β1 rise the expression of DOT1L, H3K79me2, total EGFR and improved the phosphorylation level of EGFR. EPZ5676 exhibited a dose-dependent inhibition of these responses. (Fig. 5a-d). Immunofluorescence of HPMCs demonstrated that 10μM EPZ5676 significantly reduced the 2ng/ml TGF-β1 or 60mM high glucose induced the over-expression of EGFR (Fig. 5e) and the phosphorylation of EGFR (Fig. 5f). Collectively, these findings indicate that DOT1L plays a role in mediating the expression and activation of EGFR in mesothelial cells.

### DOT1L-elicited EGFR signaling changes the phenotype of HPMCs and promotes peritoneal fibrosis of mice

**Fig. 6:**
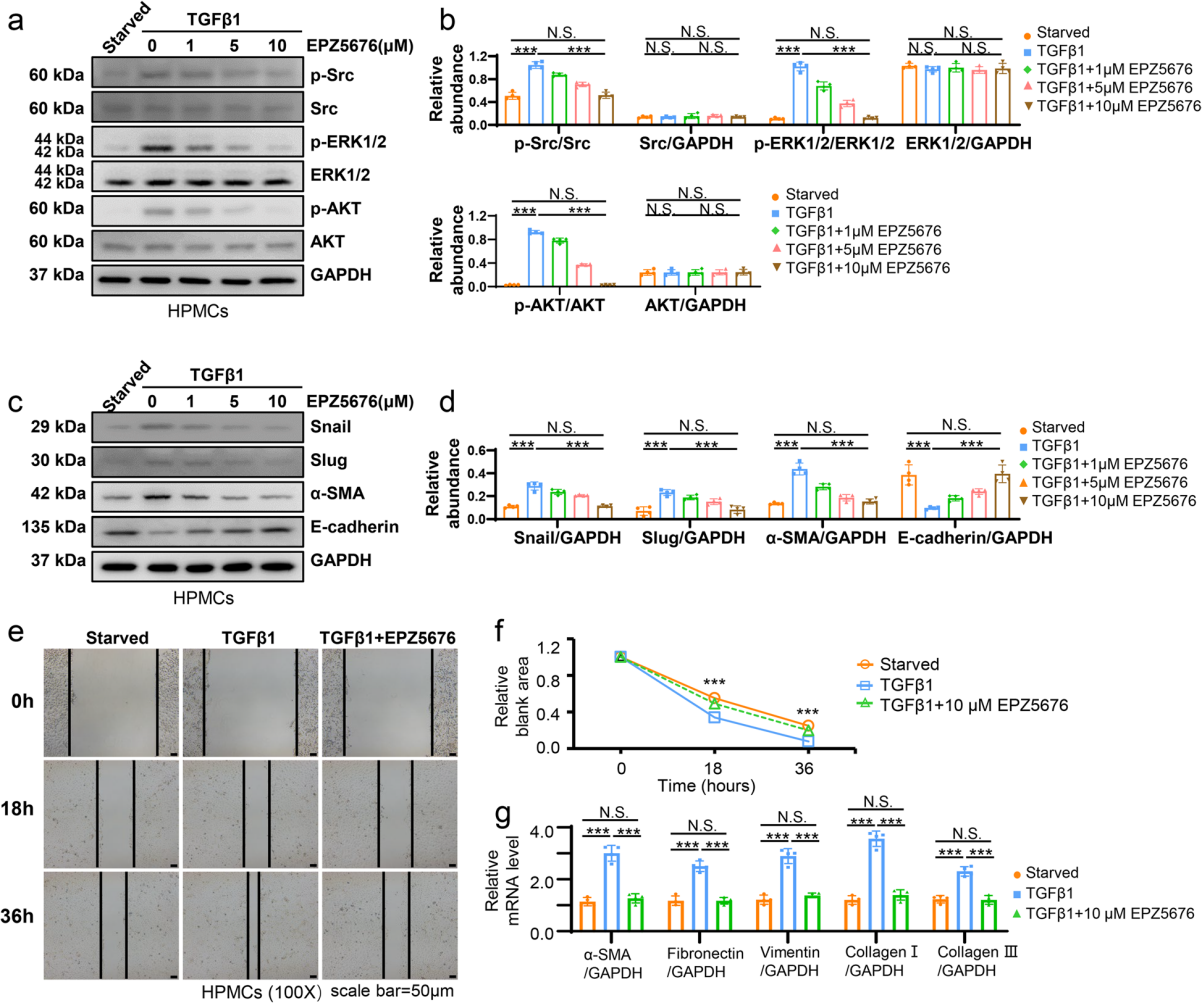
EPZ5676 inhibits EGFR related or downstream signaling pathway and prevents the phenotype change of HPMCs. **a** Serum-starved HPMCs were cultured in 2ng/ml TGF-β1 for 36 h with different concentrations of EPZ5676 (0, 1, 5, 10 μM). Cell lysates were subjected to immunoblotting analysis with specific antibodies against p-Src, Src, p-ERK1/2, ERK1/2, p-AKT, AKT or GAPDH. **b** Expression levels of p-Src, p-ERK1/2 and p-AKT were quantified by densitometry and normalized with Src, ERK1/2 and AKT, respectively. Expression levels of Src, ERK1/2 and AKT were quantified by densitometry and normalized with GAPDH. **c** Cell lysates were subjected to immunoblotting analysis with specific antibodies against Snail, Slug, α-SMA, E-cadherin and GAPDH. **d** Expression levels of Snail, Slug, α-SMA and E-cadherin were quantified by densitometry and normalized with GAPDH. **e** Wound-healing assay of HPMCs treated with TGF-β1 (2 ng/ml) in the presence or absence of EPZ5676 (10 μM). Photomicrographs of migrating cells were taken at 0, 18 and 36 h. **f** The width of the wound was measured, and the migratory rate was calculated. **g** The mRNA level of α-SMA, Fibronectin, Vimentin, Collagen type I, III was tested by RT-qPCR in HPMCs with three different treatments. Scale bar = 50μm. Data are means ± sem of 4 samples, *P < 0.05, **P < 0.01, *** *P* < 0.001, *P* ≥ 0.05 is not significant (NS)

Phosphorylated EGFR could co-activate intracellular Src signaling and downstream extracellular signal regulated kinase 1/2 (ERK1/2) and protein kinase B (AKT) pathway, which were thought to have crucial functions in cellular mesenchymal transition [35, 36]. We supposed that DOT1L-activated EGFR triggered these signaling cascades and contributed to phenotypic change of HPMCs. Accordingly, we used various concentrations of EPZ5676 (0, 1, 5,10 μM) to treat the damaged HPMCs and tested the expression levels of p-Src, p-ERK1/2 and p-AKT after 36h. Stimulation of HPMCs with 2 ng/ml TGF-β1 led to elevated levels of p-Src, p-ERK1/2, and p-AKT. Treatment with EPZ5676 concentrations-dependently inhibited these responses while not affecting the total protein (Fig. 6a, b). Consequently, EPZ5675 significantly decreased the TGF-β1-induced rise of Snail and Slug, two EMT-related transcription factors (Fig. 6c, d). EPZ5675 also reduced the expression of α-SMA, preserved the E-cadherin, one of the selected adhesion molecules, in a dose dependent way and obtained the best treatment effect at 10 μM (Fig. 6c, d). Wound healing assay demonstrated that TGF-β1 prominently facilitated the proliferation and migratory ability of HPMCs, which can be inhibited by 10 μM EPZ5676 (Fig. 6e, f). Moreover, qPCR detected the decreased RNA level of α-SMA, Vimentin, Fibronectin, Collagen in HPMCs after DOT1L inhibition (Fig. 6g). These results were also verified by 60 mM HG-elicited EMT of HPMCs treated by DOT1L siRNA (Figs. S2, S3 and S4). In conclusion, these findings demonstrated that DOT1L facilitated the activation of EGFR signaling and altered HPMCs phenotype.

To further examine the impact of DOT1L on the expression and activation of EGFR in vivo, we established the mouse model of PF injured by PDF/CG. Immunoblot analysis revealed that the levels of DOT1L and H3K79me2 were low in the sham peritoneum, with or without the administration of EPZ5676. However, the expression of both notably increased following the injection of PDF or CG. EPZ5676 treatment significantly inhibited the DOT1L expression and its histone substrate H3K79me2 in the peritoneum of mice (Figs. S1f, g and S5a, b). Immunofluorescent showed that DOT1L co-stained with EGFR in the fibrotic peritoneum, which suggested that extranuclear DOT1L could interact with EGFR (Fig. S5c). Immunoblotting showed that blocking DOT1L with EPZ5676 resulted in a significant decrease in EGFR phosphorylation, along with a 50% reduction in total EGFR levels in the injured peritoneum after injection with 4.25% PDF/0.1% CG (Figs. S1f, g and S5a, b). Furthermore, as shown in Fig. S5d, e, the levels of Src, ERK1/2 and AKT were at baseline in the peritoneum of sham-operated mice. While their expression levels were elevated in the peritoneum injured by PDF. Treatment with EPZ5676 could not suppress the expression of total level of Src, ERK1/2 and AKT, but reduce their phosphorylation. These data suggested that DOT1L participated in the regulation of the expression and activation of EGFR and promoted its downstream signaling during the peritoneal fibrosis.

EGFR signaling pathway was recognized as one of the primary signaling regulating the fibrosis [37]. Given the regulation of DOT1L on EGFR above, we postulated that DOT1L inhibition exerted the anti-fibrosis role and conducted the consequent experiments in the mouse model of PF. Immunoblotting showed that Snail and Slug, two hallmarks of EMT, were scarcely detectable in the sham peritoneum, regardless of EPZ5676 treatment. However, their expression levels substantially escalated in the peritoneum following the continuous exposure to dialysate. Administration of EPZ5676 suppressed their upregulation (Fig. S6a, b). EPZ5676 additionally suppressed the over-expressions of α-SMA and vimentin, while restoring the adhesion protein, E-cadherin, to the normal level in PDF or CG injured peritoneum (Figs. S1h, I and S6c, d). Immunohistochemistry staining demonstrated a significant reduction in collagen type I expression in the submesothelial compact zone following EPZ5676 treatment (Fig. S6e, f). Immunoblot analysis further validated the inhibitory effect of EPZ5676 on cell proliferation. This was evident from the reduced expression of proliferating cell nuclear antigen (PCNA) and Cyclin E1, two well-known markers of proliferation, in the peritoneum of mice injured by PDF (Fig. S6g, h).

### DOT1L regulates the expression and activation of JAK3 during M2 macrophages differentiation

**Fig. 7:**
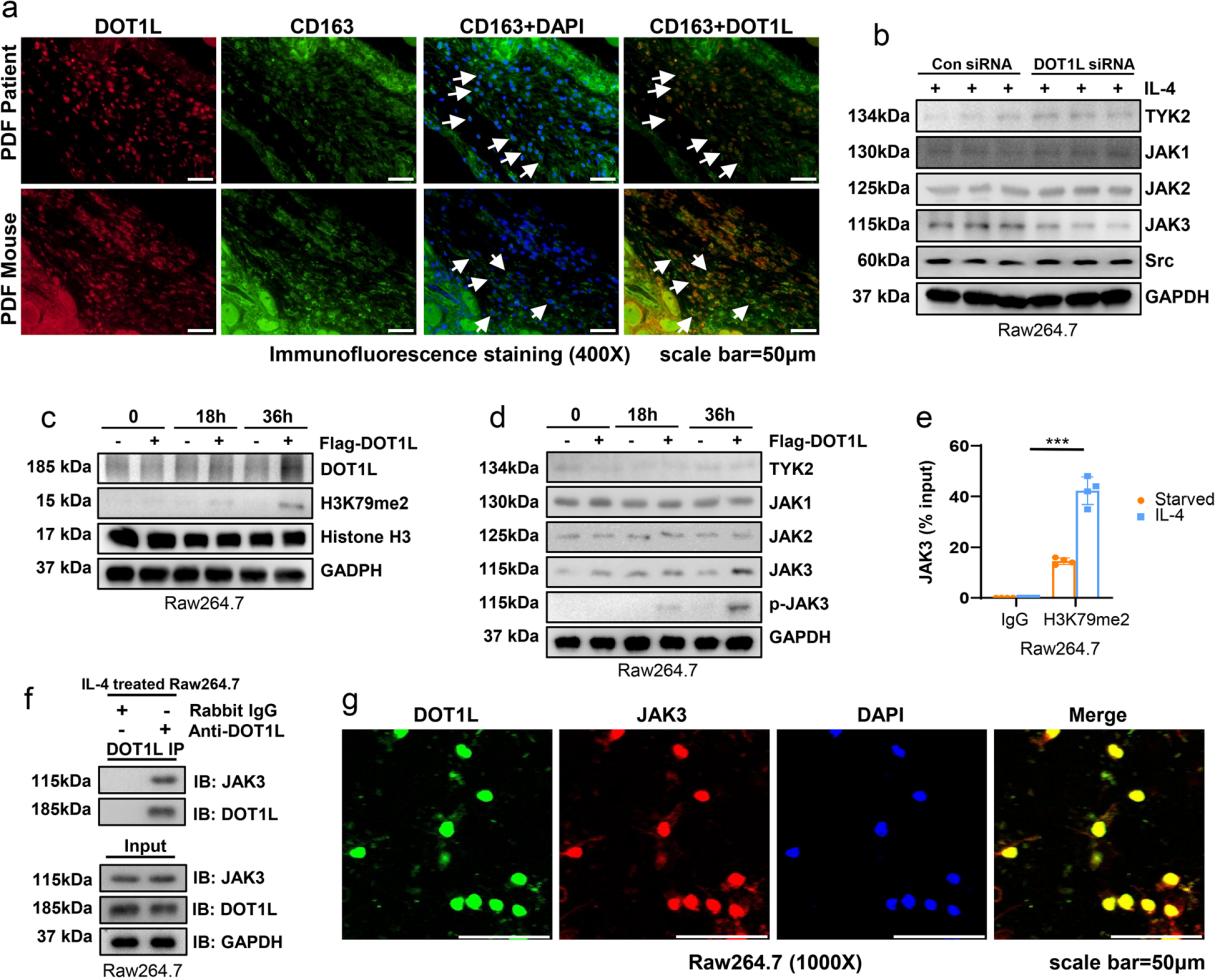
DOT1L regulates the expression and activation of JAK3 in macrophages. **a** Co-immunofluorescence photomicrographs illustrated co-staining of DOT1L and CD163 in the peritoneum from patient and mouse with peritoneal fibrosis induced by PDF. Arrows indicated CD163**-**positive cells. **b** Raw264.7 were transfected with scrambled siRNA and DOT1L siRNA for 6 h, and then incubated with 10ng/ml IL-4 for an additional 36 h before being harvested for analysis. Cell lysates were subjected to immunoblotting analysis with antibodies against TYK2, JAK1, JAK2, JAK3, Src and GAPDH. **c**, **d** Raw264.7 were transfected with Flag-DOT1L pcDNA3.0 plasmid or vector for 0, 18, 36 h and then subjected to immunoblotting analysis with antibodies against DOT1L, H3K79me2, Histone H3, TYK2, JAK1, JAK2, JAK3, p-JAK3 and GAPDH. **e** ChIP-qPCR analysis of H3K79me2 enrichment in the JAK3 promoter region. **f** 10ng/ml IL-4 treated Raw264.7 were subjected to immunoprecipitation with IgG or DOT1L antibody, followed by JAK3 and DOT1L immunoblotting. Input lysates were analyzed by JAK3, DOT1L and GAPDH immunoblotting. **g** Immunofluorescence co-staining of DOT1L and JAK3 in IL-4-treated Raw264.7

Macrophage infiltration was a common phenomenon in peritoneal fibrosis, and its M2 differentiation was an important source of cells with profibrotic phenotype [17]. Immunofluorescent staining showed that DOT1L was stained in the CD163-markered M2 macrophages (Fig. 7a). We speculated that DOT1L might participate in the signaling activation involved in M2 differentiation. Thus, we examined the expression of a variety of PTKs in IL-4-treated macrophages with or without DOT1L siRNA.

Immunoblotting results revealed that the use of DOT1L siRNA led to a decrease in JAK3 expression in IL-4-stimulated Raw264.7 cells, compared to those treated with scrambled siRNA. There was no change for other TYK2, JAK1, JAK2 and Src in the absent or present of DOT1L (Fig. 7b). Parallel to these observations, overexpression of DOT1L increased the protein levels of DOT1L and JAK3 after 18 h of DOT1L intervene, and they reached the peak at 36 h (Fig. 7c, d). ChIP qPCR analysis further demonstrated that DOT1L regulated JAK3 expression by H3K79me2 in IL-4-treated Raw264.7 (Fig. 7e). Given that DOT1L overexpression not only increased the total JAK3, but also promoted its activation, marked by autophosphorylation (Fig. 7d). We speculated that DOT1L might interact with JAK and affect its activity. To verify the hypothesis, we used an in vitro macrophages M2 differentiation model by exposure of Raw264.7 to IL-4 and DOT1L antibody to find its reciprocal protein. Immunoblot analysis detected JAK3 in the immunoprecipitated complexes with DOT1L, while it was absent in the negative control IgG complexes (Fig. 7f). Additionally, dual immunofluorescence staining revealed co-localization of DOT1L with JAK3 (Fig. 7g). These data suggest that intranuclear DOT1L regulated the expression of JAK3 and extranuclear DOT1L might interact with JAK3 and increase its activation.

To verify the regulation of DOT1L on macrophages differentiation, cultured macrophages were stimulated by 10ng/ml IL-4 and treated with DOT1L inhibitor EPZ5676 at different doses (1, 5, 10 μM). Immunoblotting indicated that IL-4 increased the expression of DOT1L, H3K79me2 and M2 markers (CD163 and Arginase-1). EPZ5676 dose-dependently suppressed these responses (Fig. S7). These results were also verified by 10ng/ml IL-4-stimulated Raw264,7 treated by DOT1L siRNA (Fig. S8). Taken together, these data demonstrated that DOT1L regulated M2 macrophages differentiation might through the protein tyrosine kinase JAK3 signaling pathway.

### Peritoneal mesothelial cells cross talk with macrophages by IL-4

**Fig. 8:**
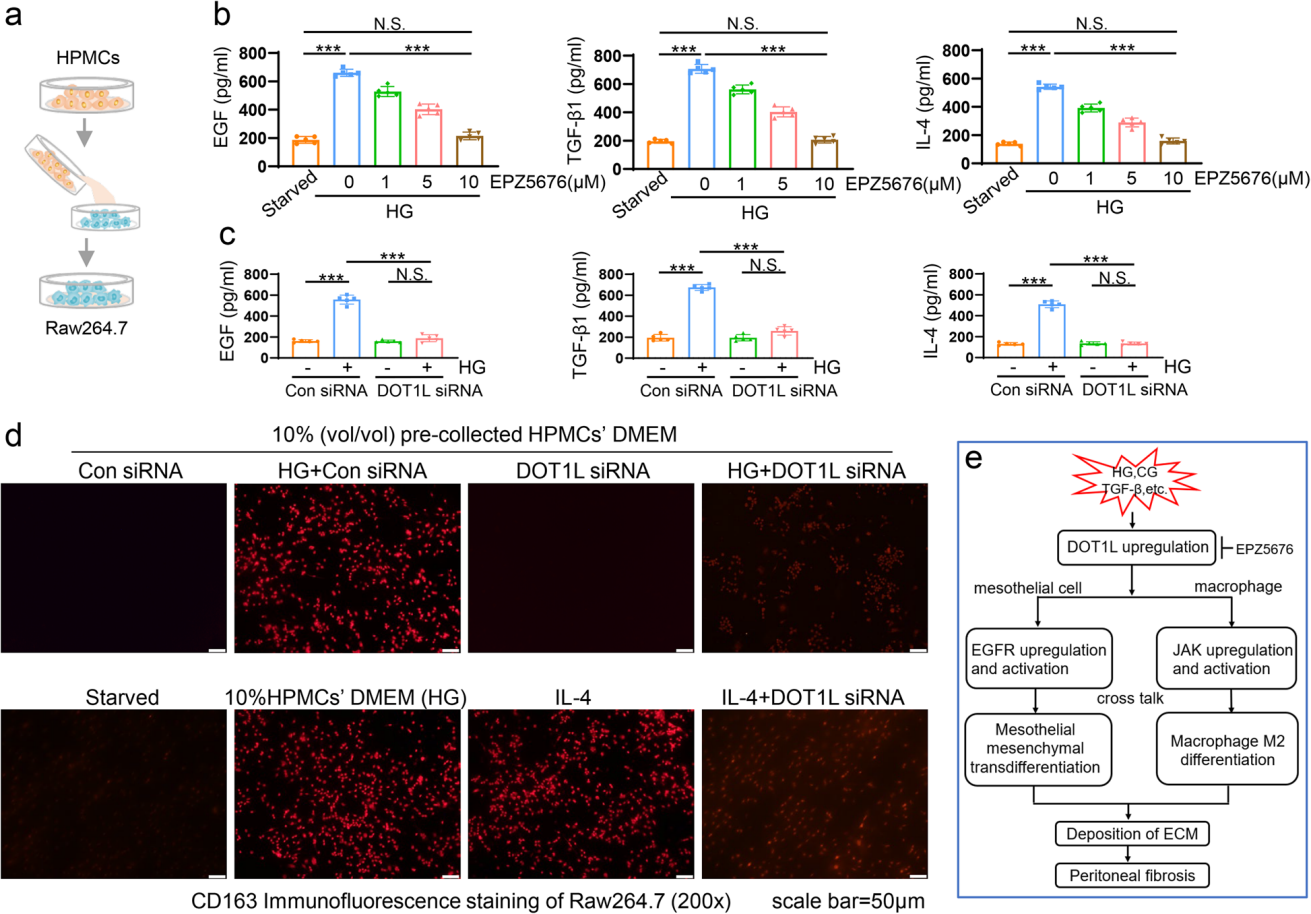
Mesothelial cells crosstalk with macrophages by secreting cytokines. **a** Raw264.7 cells were cultured with 10% (vol/vol) pre-collected cell culture media from HPMCs with different treatment. **b** Serum-starved HPMCs were cultured in 60mM high glucose for 36 h with different concentrations of EPZ5676 (0, 1, 5, 10 μM). Bar graphs showed the expression levels of EGF, TGF-β1 and IL-4 in media from HPMCs according to ELISA. **c** Serum-starved HPMCs were pretreated with scramble siRNA or DOT1L siRNA and then exposed to 60mM high glucose for an additional 36 h. Bar graphs showed the expression levels of EGF, TGF-β1 and IL-4 in media from HPMCs according to ELISA. **d** Immunofluorescence of CD163 in Raw264.7 cultured with different media. **e** Regulatory mechanism of DOT1L in peritoneal fibrosis. PD related stimulus induces DOT1L upregulation, which promotes the expression and activation of tyrosine kinases EGFR in mesothelial cells and JAK3 in macrophages, promoting cells differentiate into fibrotic phenotype, deposition of ECM and thus peritoneal fibrosis. Scale bar = 50μm. Data are means ± sem of 4 samples, *P < 0.05, **P < 0.01, *** *P* < 0.001, *P* ≥ 0.05 is not significant (NS)

To further understand the mechanism of macrophages differentiation and the relationship between mesothelial cells and macrophages, we used the HPMCs’ medium to culture Raw264.7. Initially, we exposed starved HPMCs to 60mM high glucose for 36 h with and without intervention. Following the 36-h period, we collected the culture medium of HPMCs. ELISA indicated that the secretion of EGF, TGF-β1 and IL-4 were increase significantly in HG-stimulated HPMCs, while DOT1L suppression by siRNA and inhibitor can prevent the rise of these cytokines (Fig. 8a-c). The obtained culture medium was utilized to stimulate the Raw264.7 cultured in the 1640 medium. Immunofluorescence staining revealed a significant increase in the number of CD163-positive cells after being cultured with the medium from HG-stimulated HPMCs, and the expression level of CD163 is similar with IL-4 stimulus. When cultured in the medium from HPMCs treated with DOT1L siRNA, macrophages express the less CD163 (Fig. 8d). These data suggested that HG-injured HPMCs secreted a series of cytokines, including IL-4, which would be an important messenger for macrophage differentiation.

To further examine the role of DOT1L in the macrophage M2 differentiation in vivo, we detected the M2 differentiation signaling (IL-4/JAK/STAT6) and M2 markers (CD163 and Arginase-1) in the PDF/CG peritoneum or rescued by EPZ5676 in vivo. Immunoblotting found the base levels of JAK3 and STAT6 in sham peritoneum whether or not treated with EPZ5676, and expression of both were elevated following PDF or CG injection. Administration of EPZ5676 inhibited the expression and phosphorylation of JAK3, phosphorylation of STAT6 in the peritoneum of mice under the PDF or CG injury (Figs. S9a, b and S10a, b). Immunoblotting analysis also confirmed that EPZ5676 could inhibit the expression levels of M2 phenotype biomarkers, CD163 and Arginase-1 in the PDF-injured peritoneum (Figs. S9c, d and S6c, d). Immunofluorescent showed that CD163 was barely detected in the sham peritoneum, their expression were obviously increased in peritoneum following chronic exposure to PDF. Administration of EPZ5676 suppressed their upregulation (Fig. S9e).

In summary, we suggest that PD related stimulators, such as HG, CG and TGF-β1, increase the expression DOT1L, which upregulate and active PTKs. DOT1L proceeds mesenchymal phenotype differentiation of MCs through upregulating and activating EGFR, and facilitates M2 differentiation of macrophages by upregulating and activating JAK3, subsequently promoting the deposition of ECM and subsequent peritoneal fibrosis. Pharmacological inhibition of DOT1L by EPZ5676 can block these processes and attenuate the peritoneal fibrosis (Fig. 8e).

## Discussion

Peritoneal fibrosis remains a prevalent complication for continuous PD patients, and there has been no established therapeutic strategy thus far [1, 8]. Using the pharmacological inhibition by EPZ5676, we shed light on epigenetic mechanisms of DOT1L-mediated cell phenotypic change and even PF. We firstly found the substantial increase of DOT1L expression in the peritoneum tissues and dialysis effluent from continuous PD patients, and it showed a positive correlation with injured factors (TGF-β1, MMP2 and VEGF) and a negative correlation with CA125, a biomarker for mesothelial cell mass. Secondly, RNA-sequence and proteomic analysis indicated that DOT1L was mainly involved in the process of protein tyrosine kinase binding and ECM structural constituent in the peritoneum. Thirdly, ChIP-qPCR, immunoprecipitation and immunofluorescence showed that DOT1L not only guided H3K79me2 to upregulate the expression of two tyrosine kinases (EGFR in mesothelial cells and JAK3 in macrophages) in the nucleus, but also prolonged the activated signaling of EGFR and JAK3 in the cytoplasm. Finally, this hypothesis was verified by in vitro models of HPMCs and Raw264.7, and in vivo models of mice injured by PDF and CG. Our previous study demonstrated that tyrosine kinase EGFR played a pivotal role in the PF,^15^ here we further suggested that DOT1L promoted the PF through the upregulation and activation of EGFR and JAK3.

EGFR was primarily a membrane receptor tyrosine kinase (RTK) activated by its ligands, which triggered EGFR dimerization and enhancing its intrinsic tyrosine kinase activity and leading to the subsequent autophosphorylation of tyrosine residues [38]. The phospho-tyrosine acts as docking site for SH2-containing signaling molecules, activating downstream intracellular pathways such as ERK1/2 and AKT, resulting in cell migration and proliferation [35]. Moreover, nuclear EGFR could act as a transcription co-factor, activating gene promoters and interacting with DNA-dependent protein kinase (DNA-PK) and PCNA, thereby regulating processes such as DNA synthesis, repair and replication [39]. Post-translation modifications of EGFR, such as ubiquitination, glycosylation and phosphorylation, were known to regulate the activity of EGFR [40–42]. Recently, methylation of EGFR has been documented to play a crucial role in receptor activation [43–45]. Arginine (R) methyltransferases PRMT5 could regulate EGFR methylation at R1175 and thus enhance tyrosine (Y)1173 phosphorylation [43]. PRMT1 was capable of catalyzing the methylation of R198/200 residues in the extracellular domain of EGFR, thereby enhancing its affinity for EGF and promoting EGFR homodimerization [44]. The lysine (K) methyltransferases WHSC1L1 has been reported to mono-methylate K721 in the tyrosine kinase domain of EGFR, promoting its downstream signaling in the absence of ligand [45]. In the current study, we also found that methyltransferase DOT1L could also regulate the activation of EGFR.

DOT1L is currently the sole histone lysine methyltransferase (KMT) identified to modify H3K79 methylation, a modification often associated with active gene expression [33]. Recent research showed that extracellular glucose could induce DOT1L abundance through O-GlcNAcylating DOT1L and increasing DOT1L stability in cancer cells [46]. In the case of PD, mesothelial cells were long-termly exposed to the HG microenvironment. In line with this, DOT1L was prominently detected in both the dialysate and peritoneum of continuous PD patients in the current study. We observed that DOT1L was primarily localized in the nucleus and, to a lesser extent, in the cytoplasm of HPMCs. EGFR widely exited in the plasma membrane, cytoplasm and nucleus. And they were co-located under the immunofluorescence, indicating that EGFR might interact with DOT1L, which was further verified by immunoprecipitation.

Overexpression of DOT1L led to the constant activation of EGFR, a process that occurred independently of ligand. It is possible that DOT1L-elicited EGFR methylation triggers its autophosphorylation, or methylation of EGFR protects phospho-EGFR from dephosphorylation or degradation, prolonging the signal cascades. Another question raised in this study pertains to the site where the methylation occurs. Given that DOT1L predominantly resides in the nucleus, it is possible that the EGFR methylation occurs in the nucleus, and interacts with transcription factor to enhance DNA replication and cell cycle progression. Activation and nuclear EGFR has previously been reported and considered as the resistance for EGFR inhibitor or antibodies [47]. However, it is more likely that EGFR methylation happens on the cytoplasm. Because augmented downstream cascades highly rely on the cytomembrane receptor activation, and few of nuclear EGFR can be shuttled back to the cytoplasm [48]. Evidenced by the cytoplasmic co-location of DOT1L and EGFR, EGFR may be methylated in the cytoplasm or cytomembrane, maintains the activated downstream cascades in an EGF-independent manner. Further studies are required to test the specific lysine catalytic site of EGFR.

JAK3 belongs to JAK family of non-receptor protein tyrosine kinases in immune cells [49]. Notably, IL-4-triggered JAK3/STAT6 pathway acts as a crucial role in the transcription and expression of M2-associated genes, such as Arginase 1 and CD163, responsible for the modulation of ECM remodeling [20]. Evidences showed that methylation of JAK3 promoter or ribonucleoproteins could alter the immune cell phenotype [50, 51]. In line with these researches, our study found that the promoter of tyrosine kinases, such as EGFR and JAK3, were more susceptible to methylation by intranuclear DOT1L, resulting in their transcription and accumulation. Moreover, extranuclear DOT1L could maintain the activation of protein kinases, evidenced by increased phosphorylation of EGFR in mesothelial cells and JAK3 in macrophages, after DOT1L overexpression.

The complete deletion of the DOT1L in mice resulted in lethality, indicating the benefit of DOT1L. It was documented that DOT1L was involved in of cardiogenesis, neurogenesis, lymphopoiesis and hematopoiesis during embryonic stage [52–55]. Thus, DOT1L inhibitors might not suit for pregnant women and children clinically. Additionally, Zhang et al. reported the protection of DOT1L on renal collection tube [56]. In their study, specific deletion of DOT1L in collecting duct with Aqp2 Cre increased the occupancy of HDAC2 on histone H3 and promoted the transcription of Edn1, which encoded endothelin1 and increased tubule injury. Their earlier research demonstrated that DOT1L supported the Aqp2^+^ phenotype of principal cells (PCs), responsible for water and sodium reabsorption [57]. However, deletion of DOT1L in PCs helped to increase the urine volume and decrease the urine osmolarity with relatively normal electrolyte and acid–base homeostasis in mice, indicating the disadvantage of DOT1L [57]. Recent study pointed that DOT1L had no benefit to proximal renal tubule, it promoted the EMT of proximal tubular epithelial cell, even facilitated the activation of renal fibroblast, contributing to renal fibrosis [27]. The role of DOT1L in kidney was controversial. Fortunately, there was no case of nephrotoxicity of DOT1L inhibitor reported in clinic trials (ClinicalTrials.gov identifier NCT02141828, NCT01684150, NCT03701295, NCT03724084), supporting its further application. Recently, it was also reported that DOT1L inhibition exerted the anti-fibrosis effect in pulmonary disease through the inactivation of lung fibroblast [28]. DOT1L inhibition also prevented the breast cancer progression through suppressing the EMT of breast epithelia cells [26]. Consistent with these observations, our study demonstrated that specific DOT1L inhibitor EPZ5676 exerted an anti-fibrosis role in peritoneum of long-term PD mice. Intriguingly, the profibrotic role of DOT1L was similar with another KMT enhancer of zeste homolog 2 (EZH2) reported in our previous studies [58–61]. Notably, DOT1L makes profibrotic gene available, while EZH2-catalyzed H3K27me3 tends to form a condensed chromatin structure, resulting in the suppression of gene expression rather than its enhancement. Therefore, we suggested that EZH2 might silent the anti-fibrosis genes, exerting the synergistic effect with DOT1L-activated profibrotic genes.

Of all the KMTs, EZH2 inhibitor are being tested to treat a series of cancers, and has come into phase III (NCT04224493) that will hopefully soon be available in clinical settings to help patients. Following that, another KMT DOT1L inhibitor EPZ5676 is also regarded as a promising drug, which has come into phase II (NCT03701295). Recent disclosing showed that EPZ5676 was generally safe, with the maximum tolerated concentration not being reached for adult acute leukemia [29]. The most frequently reported adverse events included nausea, constipation, fatigue and febrile neutropenia, which were well tolerated [29]. We look forward the further clinical investigation of EPZ5676 on PD-related peritoneal fibrosis as a stand-alone therapy or combination approach with biocompatible dialysate.

This study has a few limitations that need to be mentioned. First, the current study focuses on mesothelial cells and macrophage. Lacking the single-cell sequencing of peritoneal tissue, which helps to clarify the other contributory cells. Secondly, the clinical sample of PDF patients was from a single center, not multi-centers. Nonetheless, the research conclusion remains reliable through transcriptomics, proteomics and animal experiments. In summary, the current study uncovered a new role for DOT1L in modulating the pathophysiology of peritoneal fibrosis. Targeting DOT1L pharmacologically could potentially offer a promising therapeutic strategy for mitigating peritoneal fibrosis and improving peritoneal functions in patients undergoing PD.

## Materials and methods

Additional details for all methods were provided in the supplementary Materials.

### Clinical sample collection and ethics statement

Human PD effluent from patients undergoing dialysis of different durations were collected at Shanghai East Hospital affiliated with Tongji University from August 2017 to October 2022. These patients were divided into three groups according to duration: duration < 12 months (*n* = 29), 12 months ≤ duration < 36 months (*n* = 25), 36 months ≤ duration (*n* = 18) (Supplemental Table 1). To detect the expression of DOT1L in the peritoneum samples of PD patients, we conducted immunofluorescence staining of peritoneal tissue from long-term PD patient (36 months ≤ duration) who accepted catheter removal operations at Shanghai East Hospital affiliated with Tongji University.

### Statistical analyses

We repeated all experiments a minimum of three times. The data presented in the graphs represent the mean ± SEM for each group. Intergroup comparisons were performed using one-way analysis of variance (ANOVA). Multiple comparisons among means were conducted using Tukey’s test, while differences between two specific groups were assessed using Student’s t-test. Statistically significant differences between mean values are indicated in the respective graphs. The *P*-value < 0.05 was considered significant. Statistical analyses were carried out using IBM SPSS Statistics 20.0 (Version X; IBM, Armonk, NY, USA).

## Supplementary Information

**Additional file 1: Supplementary Fig. 1.** EPZ5676 attenuates the peritoneal fibrosis induced by CG in the mice. **Supplementary Fig. 2.** DOT1L siRNA inhibits the expression and activation of EGFR in high glucose-stimulated HPMCs. **Supplementary Fig. 3.** DOT1L siRNA inhibits EGFR related and downstream signaling pathway in high glucose-stimulated HPMCs. **Supplementary Fig. 4.** DOT1L siRNA attenuates phenotypic change of HPMCs after high glucose injury. **Supplementary Fig. 5.** EPZ5676 reduces the expression and activation of EGFR and inhibits its signaling pathway in the development of peritoneal fibrosis of mice. **Supplementary Fig. 6.** EPZ5676 attenuates the peritoneal fibrosis induced by PDF in the mice. **Supplementary Fig. 7.** EPZ5676 prevents the M2 phenotype differentiation of macrophages. **Supplementary Fig. 8.** DOT1L siRNA inhibits the expression and activation of JAK3 and prevents macrophage M2 differentiation after IL-4 stimulus. **Supplementary Fig. 9.** EPZ5676 attenuates the macrophage M2 differentiation in the mice peritoneum injured by PDF. **Supplementary Fig. 10.** EPZ5676 attenuates the macrophage M2 differentiation in the mice peritoneum injured by CG. **Supplementary Table 1.** Clinical characteristics of peritoneal dialysis patients. **Supplementary Table 2.** Details of primary antibodies used for immunoblotting analysis. **Supplementary Table 3.** Details of primary antibodies used for immunofluorescence and immunohistochemical staining. **Supplementary Table 4. **Details of second antibodies used for immunofluorescence staining. **Supplementary Table 5.** Primer sequence for real time quantitative PCR (RT-qPCR). **Supplementary Table 6.** Sequence of siRNA. **Supplementary Table 7.** Primer sequence chromatin immunoprecipitation (ChIP) assays. Supplementary Methods.

## Data Availability

The experimental data sets generated and/or analyzed during the current study are available from the corresponding author upon reasonable request. No applicable resources were generated during the current study.
