# Correction: The disruptor of telomeric silencing 1-like (DOT1L) promotes peritoneal fibrosis through the upregulation and activation of protein tyrosine kinases

**DOI:** 10.1186/s43556-026-00484-7

**Published:** 2026-06-05

**Authors:** Min Tao, Yingfeng Shi, Hui Chen, Jinqing Li, Yi Wang, Xiaoyan Ma, Lin Du, Yishu Wang, Xinyu Yang, Yan Hu, Xun Zhou, Qin Zhong, Danying Yan, Andong Qiu, Shougang Zhuang, Na Liu

**Affiliations:** 1 Department of Nephrology, Pudong New District, Shanghai East Hospital, Tongji University School of Medicine, 150 Jimo Road, Shanghai, 200120 China; 2 Shanghai Key Laboratory of Maternal and Fetal Medicine, Clinical and Translational Research Center of Shanghai First Maternity & Infant Hospital, School of Life Sciences and Technology, Tongji University, Shanghai, China; 3 Department of Medicine, Rhode Island Hospital and Alpert Medical School, Brown University, Providence, RI USA

**Correction: Mol Biomed 5, 3 (2024)**

**https://doi.org/10.1186/s43556-023-00161-z**

Following the publication of the original article [1], it was reported that in Fig. 5e, the staining image of the starved group was partially overlapped with the starved + EPZ5676 group. This was caused by a negligent layout of images in the control groups (starved with/without medication). The starved + EPZ5676 group has now been replaced with the correct image. The updated figure does not affect the study’s results or conclusions.

Fig. 5 has been corrected from:

**Fig. 5:**
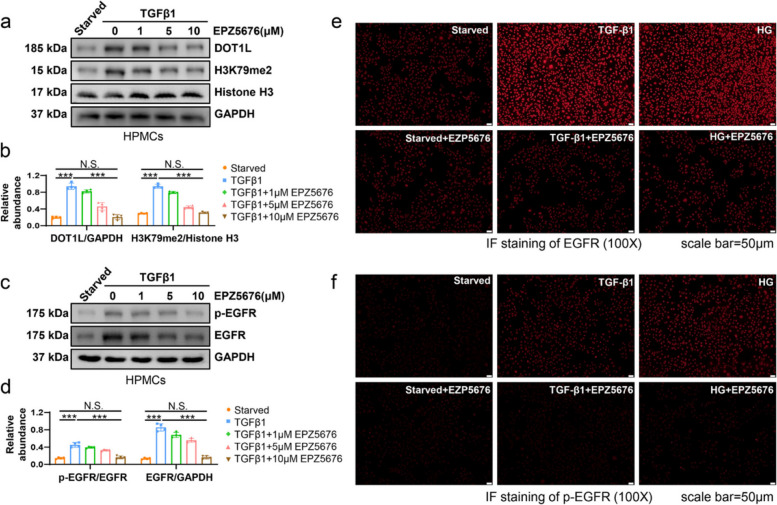
DOT1L inhibitor EPZ5676 reduces the expression and activation of EGFR in peritoneal mesothelial cells. **a** Serum-starved HPMCs were cultured in 2 ng/ml TGF-β1 for 36 h with different concentrations of EPZ5676 (0, 1, 5, 10 μM). Cell lysates were subjected to immunoblotting analysis with specific antibodies against DOT1L, H3K79me2, Histone H3 or GAPDH. **b** Expression levels of DOT1L and H3K79me2 were quantified by densitometry and normalized with GAPDH, Histone H3, respectively. **c** Cell lysates were subjected to immunoblotting analysis with specific antibodies against p-EGFR, EGFR or GAPDH. **d** Expression levels of p-EGFR and EGFR were quantified by densitometry and normalized with EGFR and GAPDH, respectively. **e** Immunofluorescence of EGFR in HPMCs under different treatments (starved, 2 ng/ml TGF-β1 or 60 mM HG) with or without 10 μM EPZ5676. **f** Immunofluorescence of p-EGFR in HPMCs under different treatments with or without 10 μM EPZ5676. Scale bar = 50 μm. Data are means ± SEM of 4 samples, *P < 0.05, **P < 0.01, *** *P* < 0.001, *P* ≥ 0.05 is not significant (NS)

To:

**Fig. 5:**
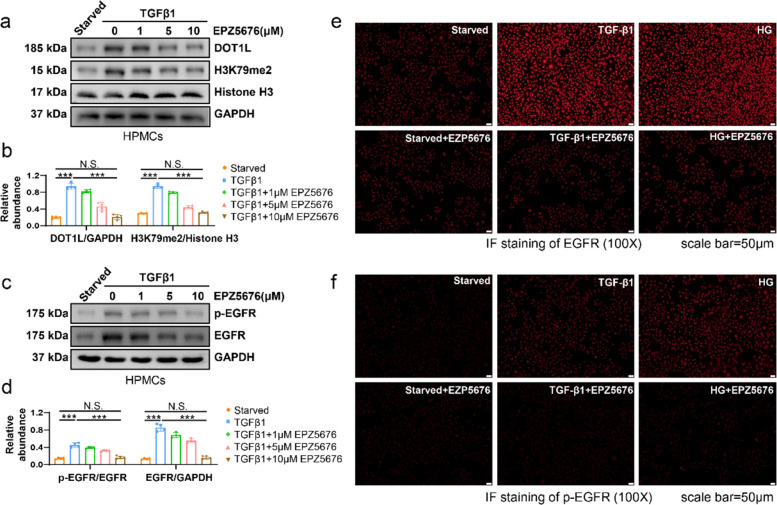
DOT1L inhibitor EPZ5676 reduces the expression and activation of EGFR in peritoneal mesothelial cells. **a** Serum-starved HPMCs were cultured in 2 ng/ml TGF-β1 for 36 h with different concentrations of EPZ5676 (0, 1, 5, 10 μM). Cell lysates were subjected to immunoblotting analysis with specific antibodies against DOT1L, H3K79me2, Histone H3 or GAPDH. **b** Expression levels of DOT1L and H3K79me2 were quantified by densitometry and normalized with GAPDH, Histone H3, respectively. **c** Cell lysates were subjected to immunoblotting analysis with specific antibodies against p-EGFR, EGFR or GAPDH. **d** Expression levels of p-EGFR and EGFR were quantified by densitometry and normalized with EGFR and GAPDH, respectively. **e** Immunofluorescence of EGFR in HPMCs under different treatments (starved, 2 ng/ml TGF-β1 or 60 mM HG) with or without 10 μM EPZ5676. **f** Immunofluorescence of p-EGFR in HPMCs under different treatments with or without 10 μM EPZ5676. Scale bar = 50 μm. Data are means ± SEM of 4 samples, *P < 0.05, **P < 0.01, *** *P* < 0.001, *P* ≥ 0.05 is not significant (NS)

The original article [1] has been corrected.
