# Supplementary material for: The disruptor of telomeric silencing 1-like (DOT1L) promotes peritoneal fibrosis through the upregulation and activation of protein tyrosine kinases
Source: Mol Biomed. 2024 Jan 4;5:3. doi: 10.1186/s43556-023-00161-z (PMC10764708; doi:10.1186/s43556-023-00161-z)
Supplement: Supplementary file 1 — Additional file 1: Supplementary Fig. 1. EPZ5676 attenuates the peritoneal fibrosis induced by CG in the mice. Supplementary Fig. 2. DOT1L siRNA inhibits the expression and activation of EGFR in high glucose-stimulated HPMCs. Supplementary Fig. 3. DOT1L siRNA inhibits EGFR related and downstream signaling pathway in high glucose-stimulated HPMCs. Supplementary Fig. 4. DOT1L siRNA attenuates phenotypic change of HPMCs after high glucose injury. Supplementary Fig. 5. EPZ5676 reduces the expression and activation of EGFR and inhibits its signaling pathway in the development of peritoneal fibrosis of mice. Supplementary Fig. 6. EPZ5676 attenuates the peritoneal fibrosis induced by PDF in the mice. Supplementary Fig. 7. EPZ5676 prevents the M2 phenotype differentiation of macrophages. Supplementary Fig. 8. DOT1L siRNA inhibits the expression and activation of JAK3 and prevents macrophage M2 differentiation after IL-4 stimulus. Supplementary Fig. 9. EPZ5676 attenuates the macrophage M2 differentiation in the mice peritoneum injured by PDF. Supplementary Fig. 10. EPZ5676 attenuates the macrophage M2 differentiation in the mice peritoneum injured by CG. Supplementary Table 1. Clinical characteristics of peritoneal dialysis patients. Supplementary Table 2. Details of primary antibodies used for immunoblotting analysis. Supplementary Table 3. Details of primary antibodies used for immunofluorescence and immunohistochemical staining. Supplementary Table 4. Details of second antibodies used for immunofluorescence staining. Supplementary Table 5. Primer sequence for real time quantitative PCR (RT-qPCR). Supplementary Table 6. Sequence of siRNA. Supplementary Table 7. Primer sequence chromatin immunoprecipitation (ChIP) assays. Supplementary Methods. [file 43556_2023_161_MOESM1_ESM.docx]

Supplementary Materials for

**The disruptor of telomeric silencing 1-like (DOT1L) promotes peritoneal fibrosis through the** **upregulation and activation of protein tyrosine kinases**

Min Tao^1^, Yingfeng Shi^1^, Hui Chen^1^, Jinqing Li^1^, Yi Wang^1^, Xiaoyan Ma^1^, Lin Du^1^, Yishu Wang^1^, Xinyu Yang^1^, Yan Hu^1^, Xun Zhou^1^, Qin Zhong^1^, Danying Yan^1^, Andong Qiu^2^, Shougang Zhuang^1,3^, Na Liu^1^

**Correspondence and offprint requests to: Na Liu,** M.D., Ph.D., Department of Nephrology, Shanghai East Hospital, Tongji University School of Medicine, 150 Jimo road, Pudong new district, Shanghai 200120, China. E-mail: naliubrown@tongji.edu.com.

**This PDF file includes:**

**1. Supplementary Figures**

**2. Supplementary Tables**

**3. Supplementary Methods**

1. **
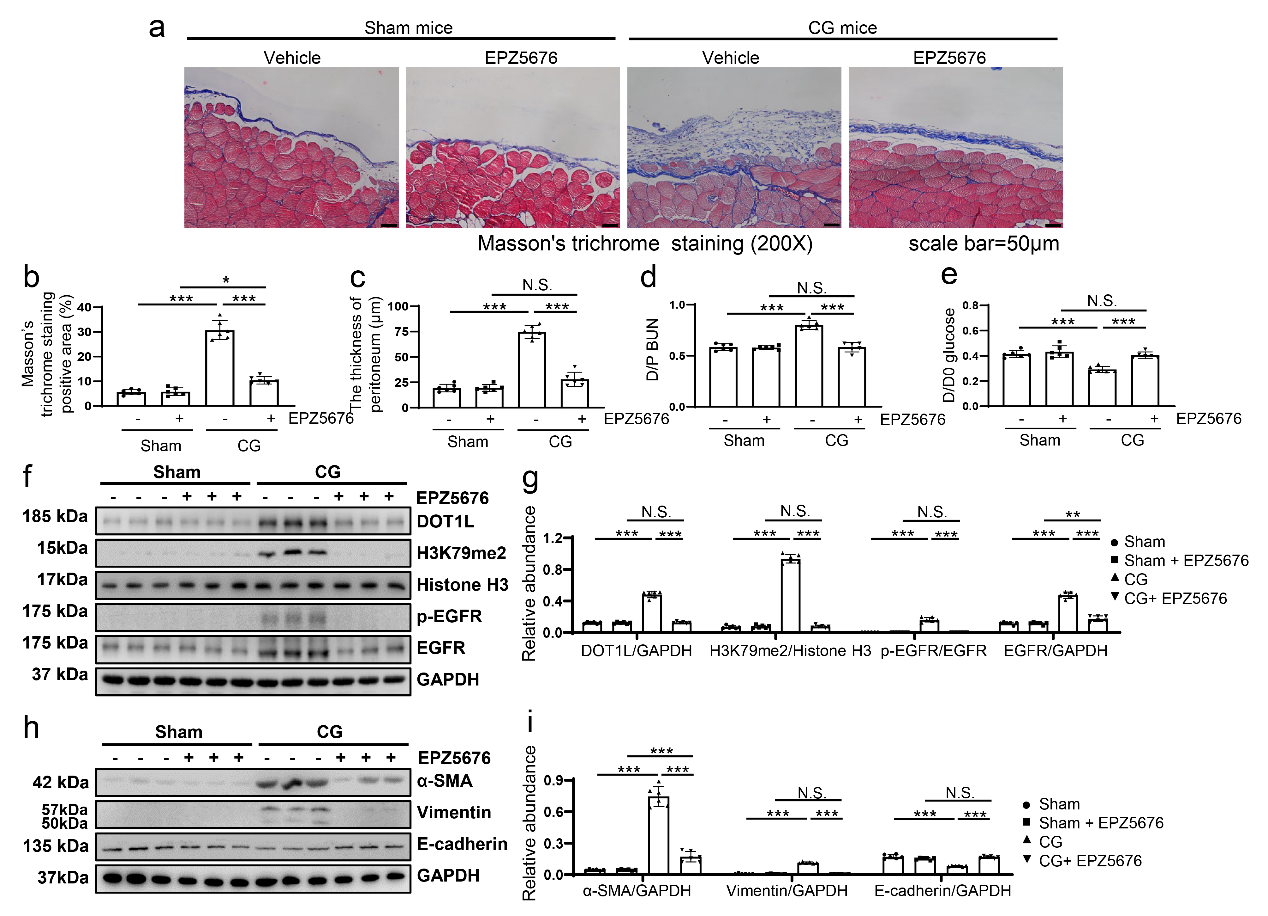
Supplementary Figures**

**Supplementary Fig.1 EPZ5676 attenuates the peritoneal fibrosis induced by CG in the mice. a** Photomicrographs showed Masson’s trichrome staining of the peritoneum in each group. **b** The bar graph showed the positive area of the Masson-positive submesothelial area (blue) from 10 random fields of peritoneal samples from six mice. **c** The bar graph showed the thickness of the compact zone measured from 10 random fields of peritoneal samples from six mice. **d** The D/P ratio of BUN. **e** The D/D0 ratio of glucose. **f** Peritoneum tissue lysates were prepared and subjected to immunoblotting analysis with antibodies against DOT1L, H3K79me2, Histone H3, p-EGFR, EGFR or GAPDH. **g** Expression levels of DOT1L, H3K79me2, p-EGFR and EGFR were quantified by densitometry and normalized with GAPDH, Histone H3, EGFR and GAPDH, respectively. **h** Peritoneum tissue lysates were subjected to immunoblotting analysis with antibodies against α-SMA, Vimentin, E-cadherin or GAPDH. **i** Expression levels of α-SMA, Vimentin and E-cadherin were quantified by densitometry and normalized with GAPDH. Scale bar=50μm. Data are means ± sem of 6 samples, **P*< 0.05, ***P* < 0.01, *** *P* < 0.001, *P* ≥ 0.05 is not significant (NS).


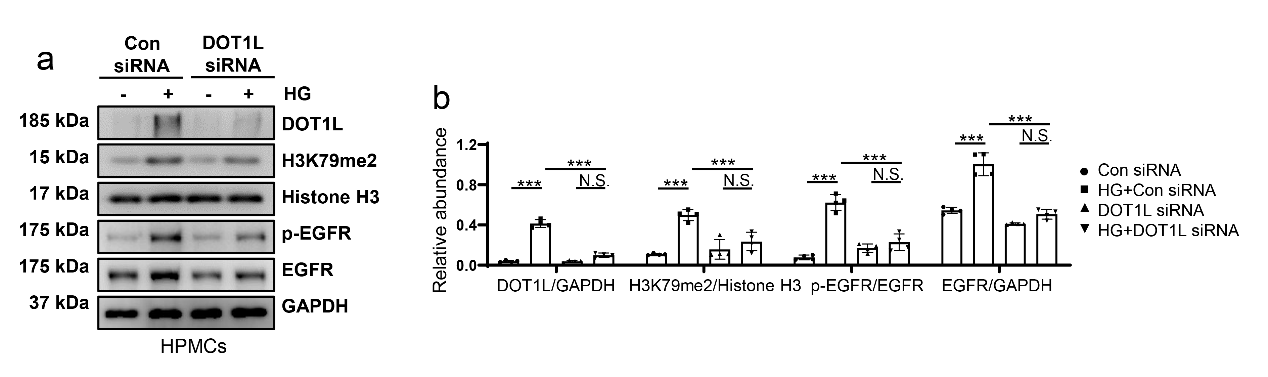


**Supplementary Fig.2 DOT1L siRNA inhibits the expression and activation of EGFR in high glucose-stimulated HPMCs.**

**a** Serum-starved HPMCs were pretreated with scramble siRNA or DOT1L siRNA and then exposed to 60mM high glucose for an additional 36 hours. Cell lysates were subjected to immunoblotting analysis with specific antibodies against DOT1L, H3K79me2, Histone H3, p-EGFR, EGFR or GAPDH. **b** Expression levels of DOT1L, H3K79me2, p-EGFR and EGFR were quantified by densitometry and normalized with GAPDH, Histone H3, EGFR and GAPDH, respectively. Data are means ± sem of 4 samples, **P*< 0.05, ***P* < 0.01, *** *P* < 0.001, *P* ≥ 0.05 is not significant (NS).


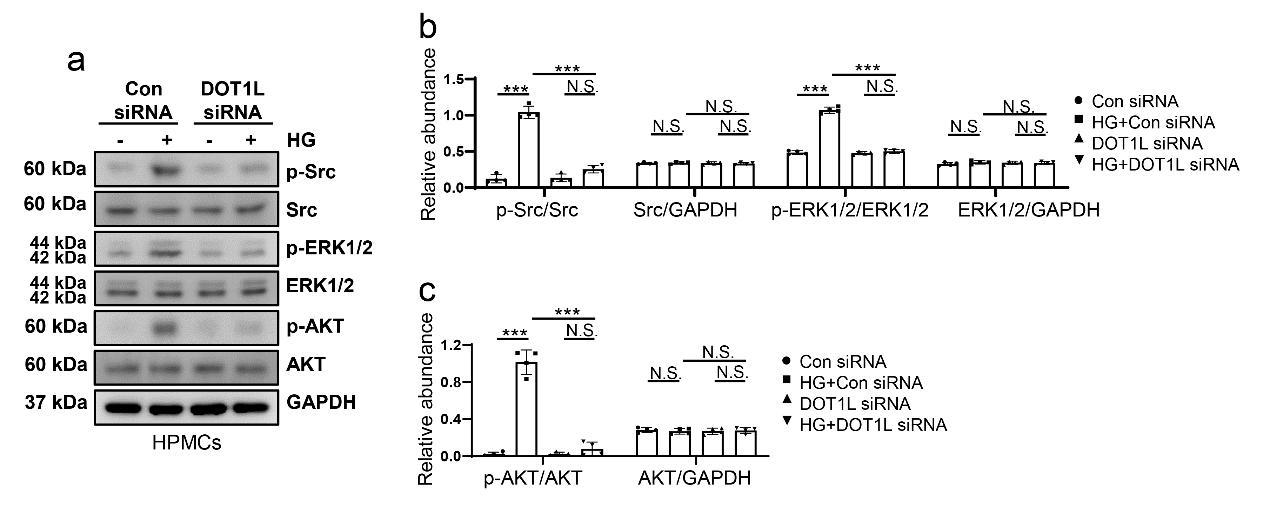


**Supplementary Fig.3 DOT1L siRNA inhibits EGFR related and downstream signaling pathway in high glucose-stimulated HPMCs.**

**a** Serum-starved HPMCs were pretreated with scramble siRNA or DOT1L siRNA and then exposed to 60mM high glucose for an additional 36 hours. Cell lysates were subjected to immunoblotting analysis with specific antibodies against p-Src, Src, p-ERK1/2, ERK1/2, p-AKT, AKT or GAPDH. **b** Expression levels of p-Src, Src, p-ERK1/2 and ERK1/2 were quantified by densitometry and normalized with Src, GAPDH, ERK1/2 and GAPDH, respectively. **c** Expression levels of p-AKT and AKT were quantified by densitometry and normalized with AKT and GAPDH, respectively. Data are means ± sem of 4 samples, **P*< 0.05, ***P* < 0.01, *** *P* < 0.001, *P* ≥ 0.05 is not significant (NS).


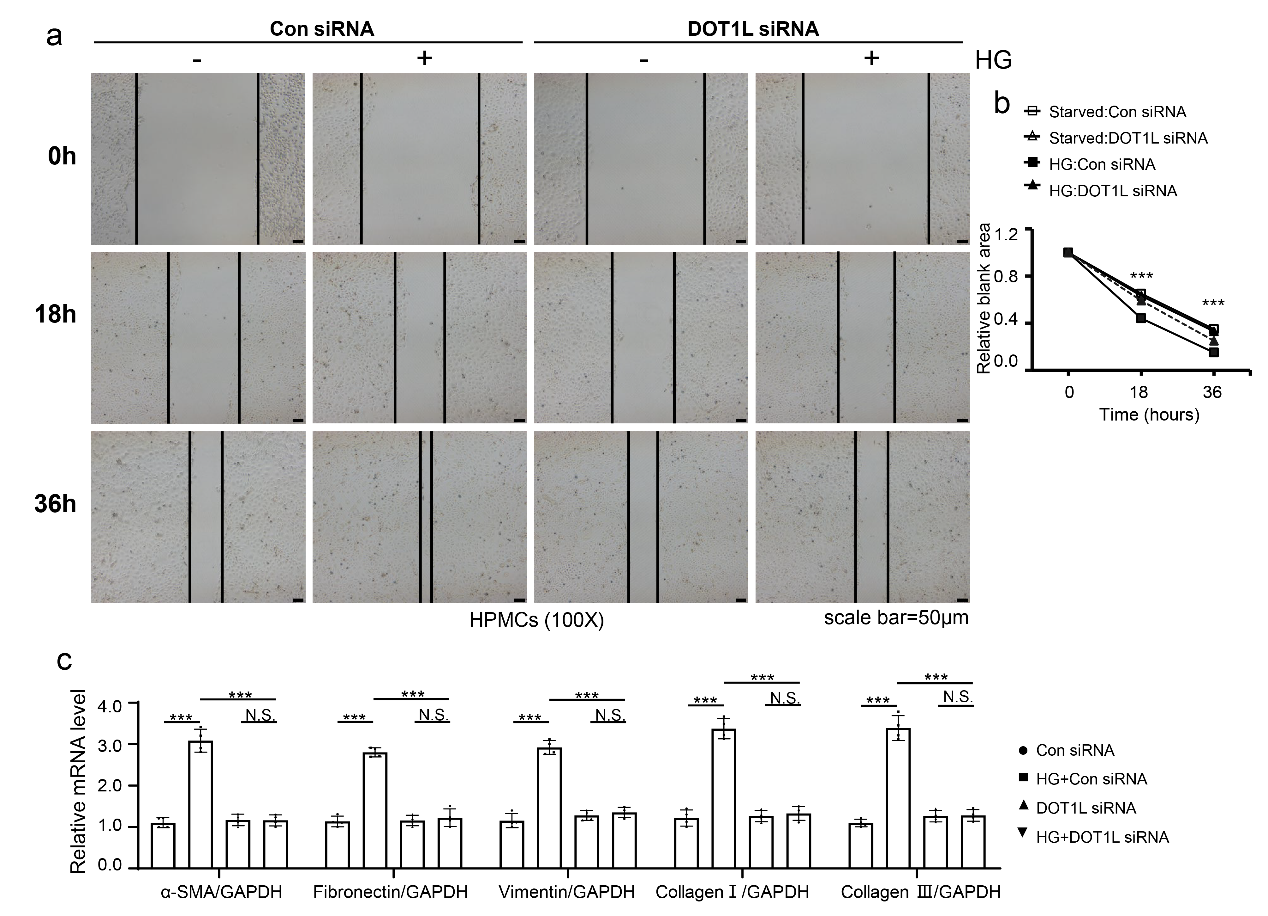


**Supplementary Fig.4** **DOT1L siRNA attenuates phenotypic change of HPMCs after high glucose injury.**

Serum-starved HPMCs were pretreated with scramble siRNA or DOT1L siRNA and then exposed to 60mM high glucose for an additional 36 hours. **a** Wound-healing assay of HPMCs treated with 60mM high glucose in the presence or absence of DOT1L. Photomicrographs of migrating cells were taken at 0 h,18h and 36 hours. **b** The width of the wound was measured, and the migratory rate was calculated. **c** The RNA level of α-SMA, Fibronectin, Vimentin and Collagen type Ⅰ and Ⅲ were tested by RT-qPCR in HPMCs with four different treatments. Scale bar=50μm. Data are means ± sem of 4 samples, **P*< 0.05, ***P* < 0.01, *** *P* < 0.001, *P* ≥ 0.05 is not significant (NS).


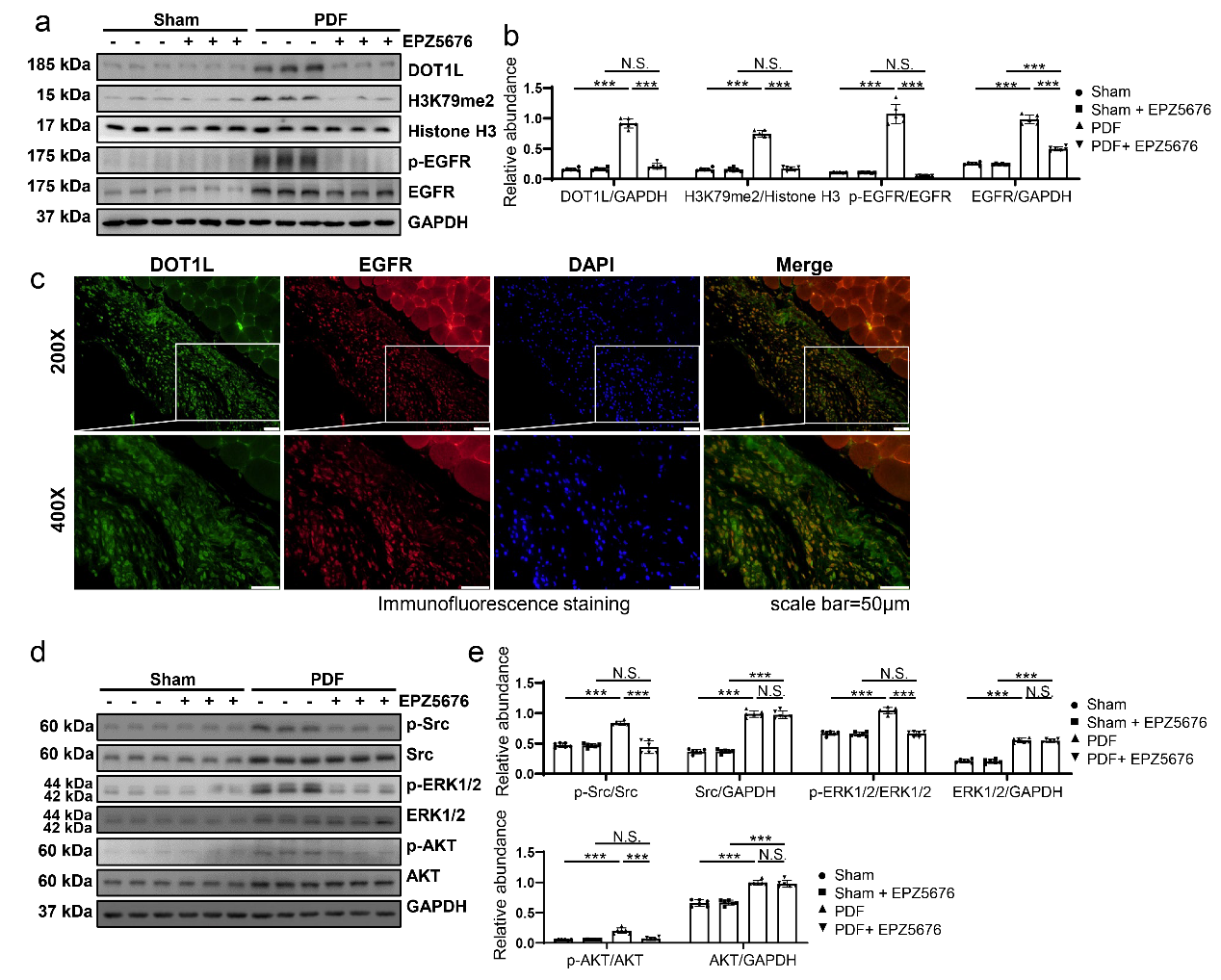


**Supplementary Fig.5 EPZ5676 reduces the expression and activation of EGFR and inhibits its signaling pathway in the development of peritoneal fibrosis of mice.** **a** Peritoneal tissue lysates were prepared and subjected to immunoblotting analysis with antibodies against DOT1L, H3K79me2, Histone H3, p-EGFR, EGFR or GAPDH. **b** Expression levels of DOT1L, H3K79me2, p-EGFR and EGFR were quantified by densitometry and normalized with GAPDH, Histone H3, EGFR and GAPDH, respectively. **c** Co-immunoﬂuorescence photomicrographs illustrate co-staining of DOT1L and EGFR in the peritoneum from mice with peritoneal fibrosis induced by high glucose PDF injection for 28 days. **d** Peritoneal tissue lysates were subjected to immunoblotting analysis with antibodies against p-Src, Src, p-ERK1/2, ERK1/2, p-AKT, AKT or GAPDH. **e** Expression levels of p-Src, p-ERK1/2 and p-AKT were quantified by densitometry and normalized with Src, ERK1/2 and AKT, respectively. Expression levels of Src, ERK1/2 and AKT were quantified by densitometry and normalized with GAPDH. Scale bar=50μm. Data are means ± sem of 6 samples, **P*< 0.05, ***P* < 0.01, *** *P* < 0.001, *P* ≥ 0.05 is not significant (NS).


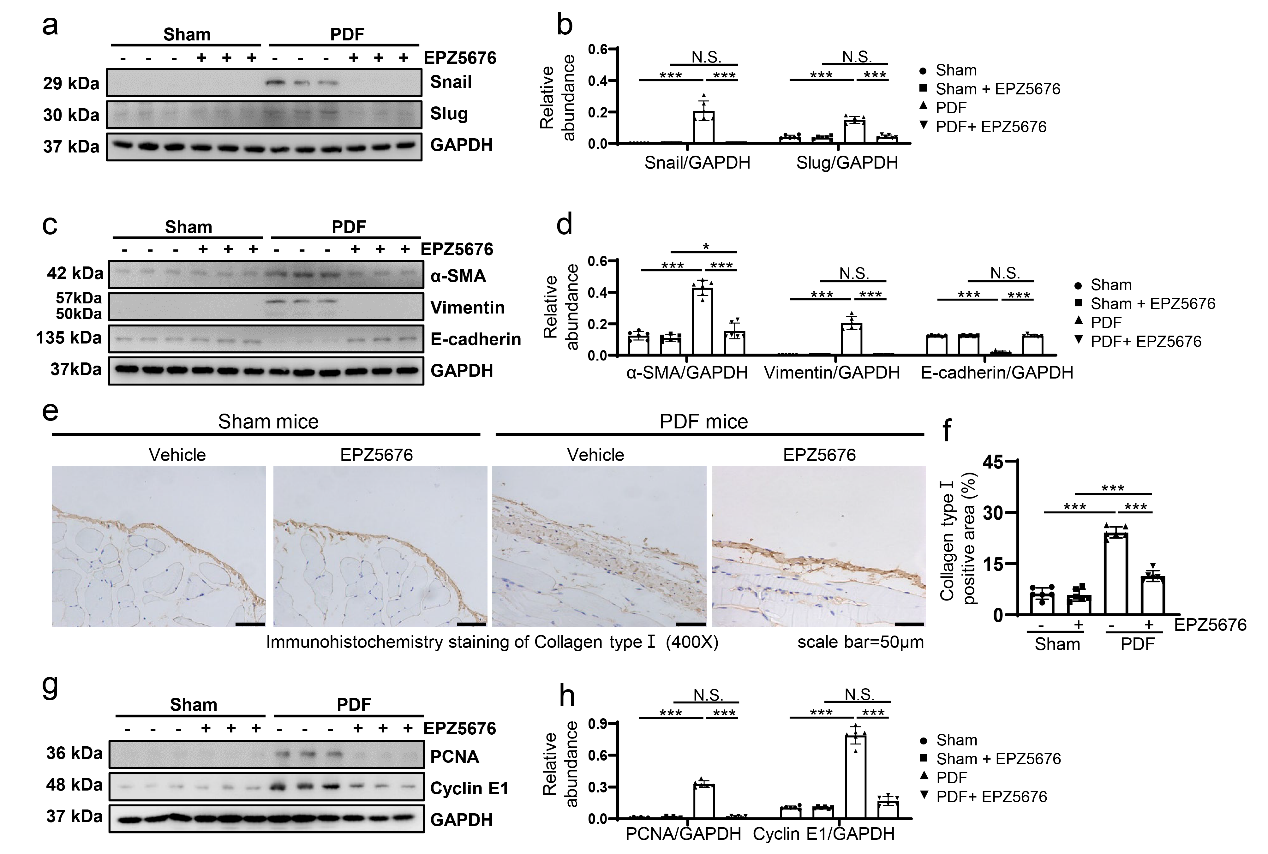


**Supplementary Fig.6** **EPZ5676 attenuates the peritoneal fibrosis induced by PDF in the mice.**

**a** Peritoneal tissue lysates were prepared and subjected to immunoblotting analysis with antibodies against Snail, Slug or GAPDH. **b** Expression levels of Snail and Slug were quantified by densitometry and normalized with GAPDH. **c** Peritoneal tissue lysates were subjected to immunoblotting analysis with antibodies against α-SMA, Vimentin, E-cadherin or GAPDH. **d** Expression levels of α-SMA, Vimentin and E-cadherin were quantified by densitometry and normalized with GAPDH. **e** Photomicrographs illustrated the immunohistochemistry staining of Collagen type I in peritoneal tissues from mice. **f** The bar graph showed the positive area of Collagen type I measured from 10 random fields of peritoneal samples from six mice. **g** Peritoneum tissue lysates were subjected to immunoblotting analysis with antibodies against PCNA, Cyclin E1 or GAPDH. **h** Expression levels of PCNA and Cyclin E1 were quantified by densitometry and normalized with GAPDH.


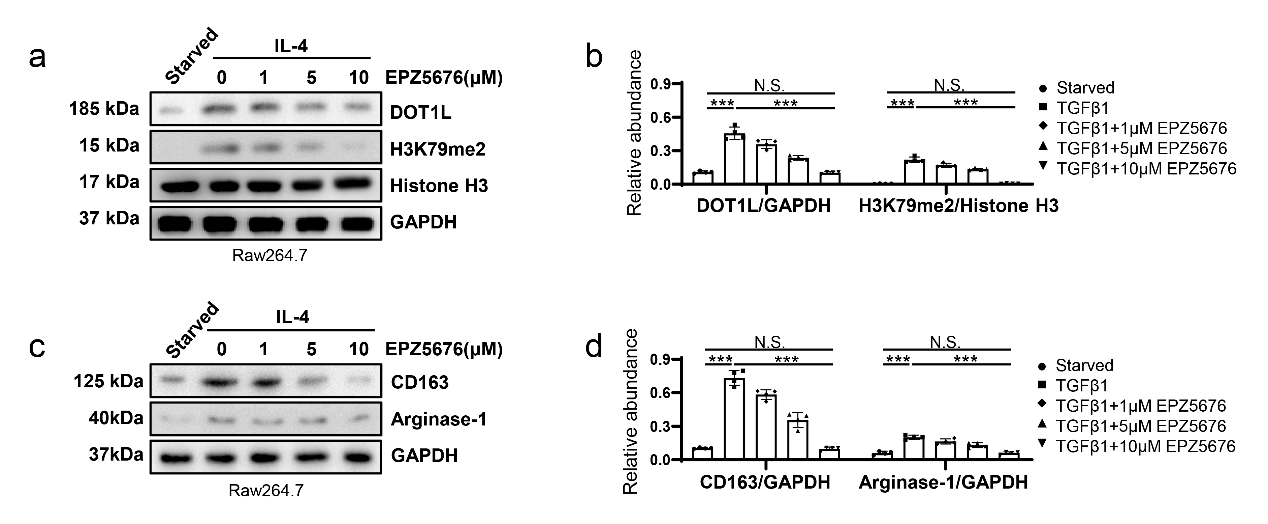


**Supplementary Fig.7 EPZ5676 prevents the M2 phenotype differentiation of macrophages.**

**a** Serum-starved Raw264.7 were cultured in 10ng/ml IL-4 for 36 hours with different concentrations of EPZ5676 (0, 1, 5, 10 μM). Cell lysates were subjected to immunoblotting analysis with specific antibodies against DOT1L, H3K79me2, Histon H3 and GAPDH. **b** Expression levels of DOT1L and H3K79me2 were quantified by densitometry and normalized with GAPDH and Histone H3, respectively. **c** Cell lysates were subjected to immunoblotting analysis with specific antibodies against CD163, Arginase-1 and GAPDH. **d** Expression levels of CD163 and Arginase-1 were quantified by densitometry and normalized with GAPDH. Data are means ± sem of 4 samples, **P*< 0.05, ***P* < 0.01, *** *P* < 0.001, *P* ≥ 0.05 is not significant (NS).


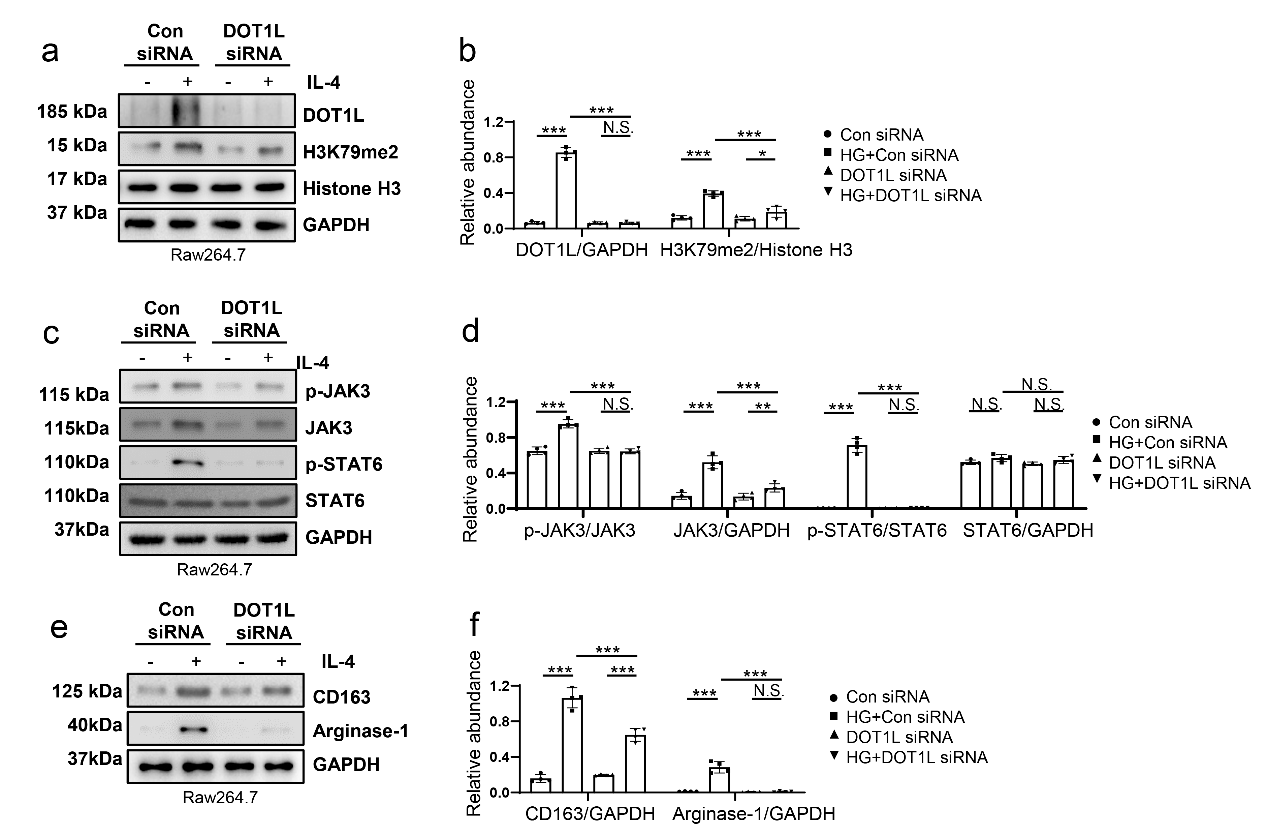


**Supplementary Fig.8 DOT1L siRNA inhibits the expression and activation of JAK3 and prevents macrophage M2 differentiation after IL-4 stimulus.**

**a** Serum-starved Raw264.7 were pretreated with scramble siRNA or DOT1L siRNA and then exposed to 10ng/ml IL-4 for an additional 36 hours. Cell lysates were subjected to immunoblotting analysis with specific antibodies against DOT1L, H3K79me2, Histone H3 or GAPDH. **b** Expression levels of DOT1L and H3K79me2 were quantified by densitometry and normalized with GAPDH and Histone H3, respectively. **c** Cell lysates were prepared and subjected to immunoblotting analysis with antibodies against p-JAK3, JAK3, p-STAT6, STAT6 or GAPDH. **d** Expression levels of p-JAK3, JAK3, p-STAT6 and STAT6 were quantified by densitometry and normalized with JAK3, GAPDH, STAT6 and GAPDH, respectively. **e** Cell lysates were subjected to immunoblotting analysis with antibodies against CD163, Arginase-1 or GAPDH. **f** Expression levels of CD163 and Arginase-1 were quantified by densitometry and normalized with GAPDH. Data are means ± sem of 4 samples, **P*< 0.05, ***P* < 0.01, *** *P* < 0.001, *P* ≥ 0.05 is not significant (NS).

**
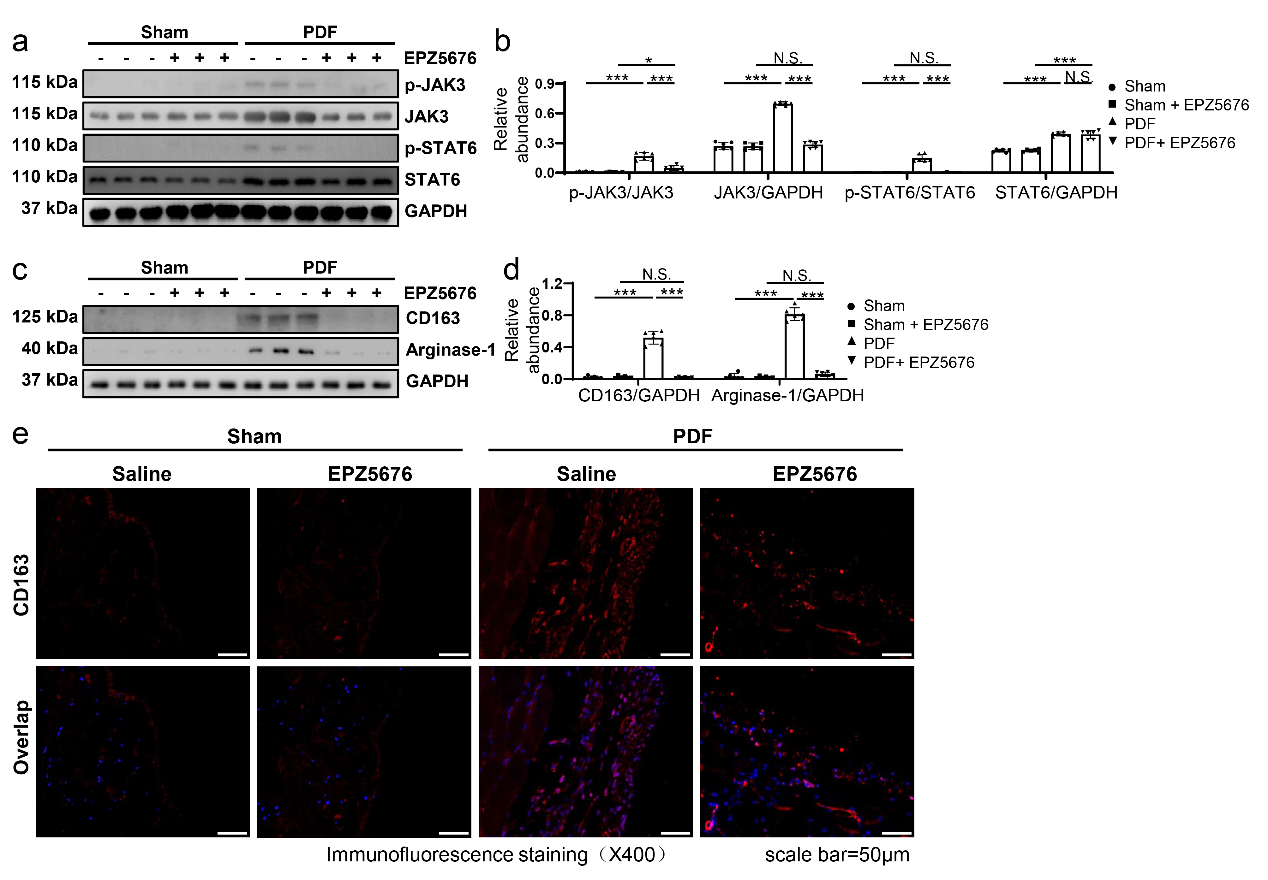
**

**Supplementary Fig.9 EPZ5676 attenuates the macrophage** **M2 differentiation in the mice peritoneum injured by PDF.**

**a** Peritoneal tissue lysates were prepared and subjected to immunoblotting analysis with antibodies against p-JAK3, JAK3, p-STAT6, STAT6 or GAPDH. **b** Expression levels of p-JAK3, JAK3, p-STAT6 and STAT6 were quantified by densitometry and normalized with JAK3, GAPDH, STAT6 and GAPDH, respectively. **c** Peritoneum tissue lysates were subjected to immunoblotting analysis with antibodies against CD163, Arginase-1 or GAPDH. **d** Expression levels of CD163 and Arginase-1 were quantified by densitometry and normalized with GAPDH. **e** Photomicrographs illustrated the immunofluorescence staining of CD163 in peritoneal tissues from mice. Scale bar=50μm. Data are means ± sem of 6 samples, **P*< 0.05, ***P* < 0.01, *** *P* < 0.001, *P* ≥ 0.05 is not significant (NS).


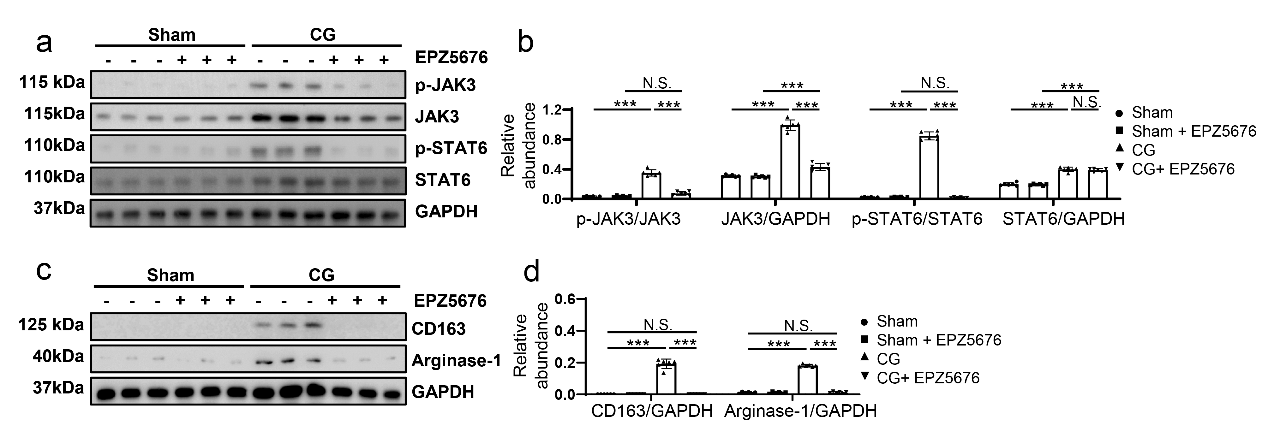


**Supplementary Fig.10 EPZ5676 attenuates the macrophage** **M2 differentiation in the mice peritoneum injured by CG.**

**a** Peritoneal tissue lysates were prepared and subjected to immunoblotting analysis with antibodies against p-JAK3, JAK3, p-STAT6, STAT6 or GAPDH. **b** Expression levels of p-JAK3, JAK3, p-STAT6 and STAT6 were quantified by densitometry and normalized with JAK3, GAPDH, STAT6 and GAPDH, respectively. **c** Peritoneal tissue lysates were subjected to immunoblotting analysis with antibodies against CD163, Arginase-1 or GAPDH. **d** Expression levels of CD163 and Arginase-1 were quantified by densitometry and normalized with GAPDH. Data are means ± sem of 6 samples, **P*< 0.05, ***P* < 0.01, *** *P* < 0.001, *P* ≥ 0.05 is not significant (NS).

1. **Supplementary Tables**

Supplementary table 1. Clinical characteristics of peritoneal dialysis patients

|  | Group Ⅰ | Group Ⅱ | Group Ⅲ | *P* value |
| --- | --- | --- | --- | --- |
|  | < 12months | 12～36months | ≥36 months |  |
| Number | 29 | 25 | 18 |  |
| PD time (months) | 3.0 (1.0-6.5) | 20.0 (14.0-26.5) | 47.0 (40.5-55.3) | <0.001 |
| Age (years) | 65.0±10.5 | 63.8±13.6 | 64.0±15.6 | 0.940 |
| Male (%) | 16 (55.2%) | 20 (80.0%) | 10 (55.6%) | 0.116 |
| Smoke (%) | 9 (31.0%) | 7 (28.0%) | 1 (5.6%) | 0.110 |
| Drink (%) | 5 (17.2%) | 2 (8.0%) | 0 (0.0%) | 0.143 |
| BMI (kg/m^2^) | 23.9±3.5 | 24.6±3.8 | 26.5±3.3 | 0.052 |
| Hemoglobin (g/L) | 99.5±16.6 | 95.6±17.0 | 94.3±13.4 | 0.492 |
| Serum albumin (g/L) | 33.1±3.9 | 32.9±4.6 | 31.2±4.3 | 0.326 |
| FBG (mmol/L) | 6.5±2.2 | 7.6±3.8 | 5.2±1.2 | 0.018 |
| ALT (U/L) | 12.0 (9.3-17.0) | 13.0 (8.5-31.5) | 13.0 (12.0-18.3) | 0.437 |
| AST (U/L) | 13.7 (11.0-18.5) | 17.0 (10.0-24.7) | 16.5 (13.0-21.7) | 0.451 |
| TB (μmol/L) | 6.3±2.9 | 8.4±6.5 | 5.9±2.8 | 0.137 |
| TC (mmol/L) | 3.7 (3.1-4.5) | 4.4 (3.7-4.8) | 3.7 (3.1-4.9) | 0.362 |
| TG (mmol/L) | 2.0 (1.4-3.1) | 1.4 (1.1-2.9) | 1.6 (1.0-2.5) | 0.146 |
| HDL-C (mmol/L) | 0.8 (0.6-1.1) | 1.0 (0.8-1.3) | 1.0 (0.8-1.2) | 0.134 |
| LDL-C (mmol/L) | 2.0 (1.5-2.6) | 2.3 (2.0-3.0) | 2.3 (1.5-3.5) | 0.283 |
| Serum UA (μmol/L) | 396.6±108.0 | 397.6±81.5 | 375.3±73.1 | 0.684 |
| Cr (μmol/L) | 894.6±314.0 | 843.3±329.8 | 771.7±252.2 | 0.412 |
| BUN (mmol/L) | 17.2±4.6 | 17.8±5.4 | 17.1±7.4 | 0.900 |
| Cystatin C (mg/L) | 6.6±1.5 | 7.3±1.6 | 6.1±1.3 | 0.056 |
| Sodium (mmol/L) | 140.5±2.5 | 140.4±3.0 | 139.8±2.2 | 0.673 |
| Potassium (mmol/L) | 3.8±0.5 | 3.7±0.6 | 3.9±0.7 | 0.439 |
| Calcium (mmol/L) | 2.3±0.2 | 2.3±0.3 | 2.2±0.2 | 0.325 |
| Phosphorus (mmol/L) | 1.6±0.5 | 1.5±0.5 | 1.5±0.5 | 0.445 |
| Total Kt/V | 2.0 (1.7-2.3) | 1.7 (1.5-1.9) | 1.7 (1.4-1.8) | <0.001 |
| Residual eGFR (ml/min/1.73m^2^) | 2.8 (1.2-4.5) | 0.7 (0.0-2.0) | 0.0 (0.0-0.5) | <0.001 |
| 4h Cr D/P | 0.7 (0.6-0.8) | 0.7 (0.6-0.8) | 0.8 (0.6-0.9) | 0.130 |
| 4h glucose D/P | 0.4 (0.4-0.5) | 0.4 (0.4-0.5) | 0.3 (0.3-0.5) | 0.309 |
| Peritoneal transport function |  |  |  | 0.215 |
| High transport (%) | 3 (10.3%) | 2 (8.0%) | 6 (33.3%) |  |
| High mean transport (%) | 13 (44.8%) | 11 (44.0%) | 6 (33.3%) |  |
| Low mean transport (%) | 11 (37.9%) | 12 (48.0%) | 5 (27.8%) |  |
| Low transport (%) | 2 (6.9%) | 0 (0.0%) | 1 (5.6%) |  |
| Primary kidney disease |  |  |  | 0.166 |
| Hypertensive nephropathy (%) | 6 (20.7%) | 3 (12.0%) | 6 (33.3%) |  |
| Diabetic nephropathy (%) | 7 (24.1%) | 12 (48.0%) | 7 (38.9%) |  |
| Glomerulonephritis (%) | 16 (55.2%) | 10 (40.0%) | 5 (27.8%) |  |

BMI: body mass index, FBG: Fasting blood glucose, ALT: alanine aminotransferase, AST: aspartate aminotransferase, TB: total bilirubin, TC: total cholesterol, TG: triglyceride, HDL-C: high-density lipoprotein cholesterol, LDL-C: low-density lipoprotein cholesterol, Serum UA: serum uric acid, Cr: creatinine, BUN: Blood urea nitrogen, eGFR: estimated glomerular filtration rate, D/P: dialysate/plasm

Supplementary table 2. Details of primary antibodies used for immunoblotting analysis.

| Antibody | Catalogue number | Supplier | Dilution |
| --- | --- | --- | --- |
| DOT1L | #77087 | Cell Signaling Technology (Danvers, MA, USA) | 1:1000 |
| PDGFRα | #3174 | Cell Signaling Technology (Danvers, MA, USA) | 1:1000 |
| IGFRβ | #9750 | Cell Signaling Technology (Danvers, MA, USA) | 1:1000 |
| FGFR1 | #9740 | Cell Signaling Technology (Danvers, MA, USA) | 1:1000 |
| Histone H3 | #3638 | Cell Signaling Technology (Danvers, MA, USA) | 1:1000 |
| EGFR | #4267 | Cell Signaling Technology (Danvers, MA, USA) | 1:1000 |
| p-EGFR | #3777 | Cell Signaling Technology (Danvers, MA, USA) | 1:1000 |
| TYK2 | #35615 | Cell Signaling Technology (Danvers, MA, USA) | 1:1000 |
| JAK1 | #50996 | Cell Signaling Technology (Danvers, MA, USA) | 1:1000 |
| JAK2 | #3230 | Cell Signaling Technology (Danvers, MA, USA) | 1:1000 |
| JAK3 | #8863 | Cell Signaling Technology (Danvers, MA, USA) | 1:1000 |
| p-JAK3 | #5031 | Cell Signaling Technology (Danvers, MA, USA) | 1:1000 |
| Src | #36D10 | Cell Signaling Technology (Danvers, MA, USA) | 1:1000 |
| p-Src | #D49G4 | Cell Signaling Technology (Danvers, MA, USA) | 1:1000 |
| ERK1/2 | #4695 | Cell Signaling Technology (Danvers, MA, USA) | 1:1000 |
| p-ERK1/2 | #4370 | Cell Signaling Technology (Danvers, MA, USA) | 1:1000 |
| AKT | #9272 | Cell Signaling Technology (Danvers, MA, USA) | 1:1000 |
| p-AKT | #9271 | Cell Signaling Technology (Danvers, MA, USA) | 1:1000 |
| STAT6 | #5397 | Cell Signaling Technology (Danvers, MA, USA) | 1:1000 |
| p-STAT6 | #56554 | Cell Signaling Technology (Danvers, MA, USA) | 1:1000 |
| Cyclin E1 | #20808 | Cell Signaling Technology (Danvers, MA, USA) | 1:1000 |
| Snail | #3879 | Cell Signaling Technology (Danvers, MA, USA) | 1:1000 |
| Vimentin | #3932 | Cell Signaling Technology (Danvers, MA, USA) | 1:1000 |
| Arginase-1 | #93668 | Cell Signaling Technology (Danvers, MA, USA) | 1:1000 |
| DOT1L | sc-376036 | Santa Cruz Biotechnology, Inc. (Santa Cruz, CA, USA) | 1:500 |
| E-cadherin | sc-7870 | Santa Cruz Biotechnology, Inc. (Santa Cruz, CA, USA) | 1:500 |
| PCNA | sc-7907 | Santa Cruz Biotechnology, Inc. (Santa Cruz, CA, USA) | 1:500 |
| GAPDH | sc-32233 | Santa Cruz Biotechnology, Inc. (Santa Cruz, CA, USA) | 1:2000 |
| H3K79me2 | ab177184 | Abcam  (Cambridge, MA, USA) | 1:2000 |
| Slug | ab27568 | Abcam  (Cambridge, MA, USA) | 1:1000 |
| Fibronectin | ab2413 | Abcam  (Cambridge, MA, USA) | 1:400 |
| α-SMA | A2547 | Sigma-Aldrich  (St. Louis, MO, USA) | 1:1000 |
| CD163 | DF8235 | Affinity Biosciences  (Changzhou, China) | 1:1000 |

Supplementary table 3. Details of primary antibodies used for immunofluorescence and immunohistochemical staining.

| Antibody | Catalogue number | Supplier | Dilution |
| --- | --- | --- | --- |
| DOT1L | ab64077 | Abcam  (Cambridge, MA, USA) | 1:200 |
| DOT1L | sc-376036 | Santa Cruz Biotechnology, Inc. (Santa Cruz, CA, USA) | 1:200 |
| α-SMA | A2547 | Sigma-Aldrich  (St. Louis, MO, USA). | 1:400 |
| EGFR | #4267 | Cell Signaling Technology (Danvers, MA, USA) | 1:200 |
| JAK3 | sc-6932 | Santa Cruz Biotechnology, Inc. (Santa Cruz, CA, USA) | 1:200 |
| Collagen type I | sc-28654 | Santa Cruz Biotechnology, Inc. (Santa Cruz, CA, USA) | 1:200 |

Supplementary table 4. Details of second antibodies used for immunofluorescence staining.

| Antibody | Catalogue number | Supplier | Dilution |
| --- | --- | --- | --- |
| CY3-mouse | GB21301 | Servicebio，(Wuhan, China) | 1:200 |
| CY3-rabbit | GB21303 | Servicebio，(Wuhan, China) | 1:200 |
| FITC-mouse | GB22301 | Servicebio，(Wuhan, China) | 1:200 |
| FITC-rabbit | GB22303 | Servicebio，(Wuhan, China) | 1:200 |

Supplementary table 5: Primer sequence for real time quantitative PCR (RT-qPCR)

| Gene Name | Primer Sequence (5’-3’) |
| --- | --- |
| α-SMA-*homo* | Forward TGAGACTTTCAATGTCCCAGCCATG |
|  | Reverse GATGCCAGTTGTGCGTCCAGAG-3 |
| Fibronectin-*homo* | Forward AATAGATGCAACGATCAGGACA |
|  | Reverse GCAGGTTTCCTCGATTATCCTT |
| Vimentin-*homo* | Forward CCTTCGTGAATACCAAGACCTGCTC |
|  | Reverse AATCCTGCTCTCCTCGCCTTCC |
| Col1α1-homo | Forward AAAGATGGACTCAACGGTCTC |
|  | Reverse CATCGTGAGCCTTCTCTTGAG |
| Col3α1-homo | Forward GGCTACTTCTCGCTCTGCTTCATC |
|  | Reverse GCATAGGACTGACCAAGATGGGAAC |
| GAPDH-*homo* | Forward CAGGAGGCATTGCTGATGAT |
|  | Reverse GAAGGCTGGGGCTCATTT |

Supplementary table 6：Sequence of siRNA

| siRNA | Sequence (5’-3’) |
| --- | --- |
| DOT1L siRNA-*homo* | GACCUGAUUCAAGCGCAGA |
| Scramble siRNA-*homo* | UUCUCCGAACGUGUCACGUTT |
| DOT1L siRNA-*mus* | GCCUCUAGGACUAACUAAUTT |
| Scramble siRNA-*mus* | TTCTCCGAACGTGTCACGT |

Supplementary table 7: Primer sequence chromatin immunoprecipitation (ChIP) assays

| Promoter | Primer Sequence (5’-3’) |
| --- | --- |
| EGFR-*homo* | Forward TTTCGATACCCAGGACCAAGCCACAGCAGG |
|  | Reverse AATATTCTTGCTGGATGCGTTTCTGTA |
| JAK3-*mus* | Forward CCTCCCCAGCGATTGTCATT |
|  | Reverse GAAGATGTGGCTTGGGGGAA |

**3. Supplementary Methods**

**Antibodies and reagents**

EPZ5676 was purchased from Selleckchem (Houston, TX, USA). Antibodies to DOT1L were purchased from Cell Signaling Technology (Danvers, MA, USA), Abcam (Cambridge, MA, USA) and Santa Cruz Biotechnology, Inc. (Santa Cruz, CA, USA). Antibodies to PDGFRα, IGFRβ, FGFR1, H3K79me2, Histone H3, EGFR, p-EGFR, TYK2, JAK1, JAK2, JAK3, p-JAK3, Src, p-Src, ERK1/2, p-ERK1/2, AKT, p-AKT, STAT6, p-STAT6, Cyclin E1, Snail, Vimentin and Arginase-1 were purchased from Cell Signaling Technology (Danvers, MA, USA). Antibodies to Collagen type I, E-cadherin, PCNA and GAPDH were purchased from Santa Cruz Biotechnology, Inc. (Santa Cruz, CA, USA). Antibodies to Slug and Fibronectin were purchased from Abcam (Cambridge, MA, USA). Antibodies to CD163 was purchased from Affinity Biosciences (Changzhou, China). Recombination TGF-β1 and ELISA kits for DOT1L, CA125, TGF-β1, VEGF and MMP2 were purchased from Cloud-Clone (Wuhan, China). ELISA kits for EGF and IL-4 were purchased from Absin (Shanghai, China). BUN and glucose biochemical reagent kit was purchased from Nanjing Jiancheng Bioengineering Institute (Nanjing, China). The DOT1L small interfering (si) RNA was purchased from GenePharma (Shanghai, China). Flag-DOT1L plasmid was purchased from Genewiz (Shanghai, China). Lipofectamine 3000 was purchased from Invitrogen (Grand Island, NY, USA). RNA-iso Plus Reagent was purchased from TaKaRa (Beijing, China). Reverse transcription kit All-In-One 5X RT MasterMix was purchased from abmGood (Shanghai, China). qPCR kit EvaGreen 2X qPCR MasterMix was purchased from abmGood (Shanghai, China). Antibodies to α-smooth muscle actin (α-SMA), D-glucose and secondary antibodies for Western blot, and all other chemicals were purchased from Sigma-Aldrich (St. Louis, MO, USA). Additional details of primary antibodies are listed in Supplemental table 2.

**Animal models and experimental design**

Male C57BL mice (Shanghai Super-B&K Laboratory Animal Corp. Ltd, Shanghai, PR China) that weighed 20–25g were housed under a 12h light–dark cycle with food and water supplies. Two mouse models of peritoneal fibrosis were established. The first peritoneal fibrosis model was created by daily intraperitoneal injection of peritoneal dialysis solution with 4.25% glucose (100 ml/kg) (Baxter Healthcare, Guangzhou, China) for 28 days ^1^. The second peritoneal fibrosis model was established by i.p. injection of 0.1% CG (10 ml/kg) (Sigma-Aldrich, St. Louis, MO, USA) dissolved in saline every other day for 21 days ^1,2^. The sham group received an intraperitoneal injection of saline only. To investigate the effect of EPZ5676 in peritoneal fibrosis, mice were injected intraperitoneally with a single dose of EPZ5676 (35 mg/kg/day) (Selleckchem, Houston, TX, USA) in vehicle (2%DMSO+5%TWeen+saline) ^3^. Mice were randomly allocated into four groups for each model: (1) sham group: intraperitoneally injected with an equivalent amount of saline and vehicle (n = 6). (2) sham + EPZ5676 group: intraperitoneally injected with an equivalent amount of saline and EPZ5676 (35 mg/kg/day) dissolved in vehicle (n = 6). (3) PDF /CG group: intraperitoneally injected with PDF/CG and vehicle (n = 6). (4) PDF/CG + EPZ5676 group: intraperitoneally injected with PDF/CG and EPZ5676 (35 mg/kg/day) dissolved in vehicle (n = 6). Mice were killed by exsanguination under anesthesia with inhaled 5% isoflurane in room air and the parietal peritoneum was collected from each mouse for further experiments at the end of 28 days for the PDF model or 21 days for the CG model.

**Peritoneal equilibration test**

Mice in each group received a peritoneal equilibration test before euthanasia in the last day. Mice were injected with 2 ml 4.25% PDF for 2 h and then euthanized for collection of blood and dialysate. Glucose in dialysate and BUN in plasma and dialysate were determined using BUN (#C013-2-1) and glucose (F006-1-1) biochemical reagent kits according to the manufacturer’s instructions (Nanjing Jiancheng Bioengineering Institute, Nanjing, China). Functional alteration of peritoneal membrane was evaluated by the urea nitrogen transport rate from plasma with the D/P ratio of blood urea nitrogen, and the glucose absorption rate from dialysate with the ratio of D/D0.

**Morphologic studies of peritoneum**

Formalin-fixed peritoneum was embedded in paraffin and cut into 3-μm-thick sections. For evaluation of peritoneal fibrosis, Masson trichrome staining was performed according to the protocol provided by the supplier (Sigma-Aldrich). The thickness of the submesothelial tissue was measured (in μm), and the average of 10 independent measurements was calculated for each section (original magnification, ×200).

**Immunohistochemical and immunofluorescent staining**

Immunohistochemical and immunofluorescent staining were performed according to procedures described in our previous studies ^1^. For immunofluorescent staining of peritoneum, the tissue sections were rehydrated and labeled with primary antibodies, including DOT1L, EGFR, CD163 and α-SMA etc. then exposed to secondary antibodies. For immunofluorescent staining of HPMCs or Raw264.7, cells were cultured on sterile glass cover slips in 24-well plates, washed three times with PBS, and fixed with 4% paraformaldehyde for 15 minutes. The fixed cells were then washed twice with PBS, permeabilized for 10 minutes with 0.2% Triton X-100, blocked for 1 hours in 5% BSA, and incubated with the appropriate primary antibodies overnight at 4 °C. Cells were labeled for 1 hours using secondary antibodies conjugated to either FITC or CY3 at room temperature followed by three washes with PBS. Cells were then counterstained with DAPI at room temperature for 5 minutes to visualize nuclear DNA. Slide or cell images were viewed with the upright microscope (BX53, OLYMPUS, China) or confocal microscope (SP8, Leica, Germany) . Additional details of antibodies are listed in Supplemental table 3, 4.

**Immunoblotting analysis**

Immunoblotting analysis was conducted as described previously ^1^. Densitometry analysis of immunoblot result was conducted by using ImageJ software (National Institutes of Health, Bethesda, MD, USA).

**RNA analysis**

Total RNA of HPMCs was extracted by RNA-iso Plus Reagent (#9108Q, TaKaRa, Beijing, China). RNA was reverse transcribed by using All-In-One 5X RT MasterMix (G592, abmGood, Shanghai, China) according to the manufacturer’s protocol. qPCR was carried out using EvaGreen 2X qPCR MasterMix (MasterMix-S, abmGood, Shanghai, China) according to the manufacturer’s instructions. Measurements were performed in triplicate, using GAPDH as the reference gene. Real-time PCR was determined by SYBR Green I Real time quantitative PCR in a CFX96 real-time RT-PCR detection system (Bio-Rad). Primer sequences are listed in Supplemental table 5.

**ELISA analyses**

ELISAs were used to measure concentrations of DOT1L, TGF-β1, VEGF, MMP2, CA125, EGF and IL-4, which was performed in accordance with the manufacturer’s instructions from Cloud-Clone (Wuhan, Hubei, China) and Absin (Shanghai, China).

**Cell culture and treatments**

Human peritoneal mesothelial cells were purchased from Jennio biotechnology company (Guangzhou, China) and cultured in Dulbecco’s modified Eagle’s medium (DMEM) with nutrient mixture F12 containing 10% fetal bovine serum (FBS) and 1% penicillin and streptomycin stock solution in an atmosphere of 5% CO_2_ and 95% air at 37℃. After passing the primary HPMCs for three generations, we obtained a stable phenotype to start the formal experiments. To examine the treatment effect of EPZ5676 in TGF-β1-injured cells in vitro, HPMCs were starved for 24h in DMEM/F12 containing 0.5% FBS and then exposed to TGF-β1 (2 ng/ml) in the presence of EPZ5676 (0, 1, 5, and 10 μM) for 36h. Cells were harvested for immunoblotting analyses.

Mouse macrophage cells (Raw264.7) were purchased from American Type Culture Collection (Manassas, VA, USA) and cultured in RPMI-1640 containing 10% FBS, 1% penicillin, and streptomycin in an atmosphere of 5% CO_2_, and 95% air at 37 °C. To examine the effect of EPZ5676 in M2 macrophage differentiation, Raw264.7 were starved for 24h in RPMI-1640 containing 0.5% FBS and then exposed to IL-4 (10 ng/ml) in the presence of EPZ5676 (0, 1, 5, and 10 μM) for 36h. Cells were harvested for immunoblotting analyses. All of the in vitro experiments were repeated at least three times.

**Transfection of siRNA or plasmid**

The DOT1L siRNA was synthesis by GenePharma (Shanghai, China) and the sequences are listed in Supplemental table 6. The plasmid Flag-DOT1L was constructed by inserting a PCR-cloned DOT1L-gene cDNA into pcDNA3.0 vector (Genewiz, Shanghai, China) and was then verified by sequencing. Transfection of siRNA or plasmid was performed according to the manufacturer’s protocol, respectively. Briefly, Mesothelial cells or macrophages were seeded to 30-40% confluence in the antibiotic-free medium and grown followed by transfection with DOT1L siRNA (60 pmol) or DOT1L-pcDNA 3.0 plasmid using Lipofectamine 3000 (CA, USA). In parallel, scrambled siRNA (60 pmol) or plasmid was used as a control for off-target changes in the cells. After transfection, cells were cultured in the medium containing 0.5% FBS for starvation and then cells were incubated with or without irritants for an additional 36 hours before being harvested for analysis.

To investigate the crosstalk between mesothelial cells and macrophage, Raw264.7 cells were cultured with or without 10% (vol/vol) pre-collected cell culture media from HPMCs treated as described above in the presence of scrambled siRNA or DOT1L siRNA for 36 h. Then, cells were harvested for further immunofluorescent staining. All of the in vitro experiments were repeated for at least three times.

**RNA-sequencing**

Total RNA was isolated from the TGF-β1 (2 ng/ml) treated HPMCs treated with or without EPZ5676. RNA integrity was assessed using the RNA Nano 6000 Assay Kit of the Bioanalyzer 2100 system (Agilent Technologies, CA, USA). A total amount of 1 μg RNA per sample was used as input material for the RNA sample preparations. The clustering of the index-coded samples was performed on a cBot Cluster Generation System using TruSeq PE Cluster Kit v3-cBot-HS (Illumina, San Diego, CA, USA) according to the manufacturer’s instructions. After cluster generation, the library preparations were sequenced on an Illumina Novaseq platform and 150 bp paired-end reads were generated. Raw pair-end reads were mapped to the human genome (hg38) with Hisat2 (v2.0.5). Differential expression analysis of two groups was performed using the DESeq2R package (1.20.0). The resulting P-values were adjusted using the Benjamini and Hochberg’s approach for controlling the false discovery rate. Genes with an adjusted P-value <0.05 found by DESeq2 were assigned as differentially expressed.

**Proteomic analysis**

Peritoneum samples were lysed and centrifuged. 120 μg of each protein sample from supernatant was taken and the volume was made up to 100 μL with lysis buffer. The supernatant was slowly loaded to the desalting column, washed with 1 mL of washing solution for three times, and then eluted twice. Peptides were separated in analytical columns, using a linear gradient elution. The separated peptides were analyzed by Q Exactive HF-X mass spectrometer (Thermo Fisher), with ion source of Nanospray Flex™(ESI), spray voltage of 2.3 kV and ion transport capillary temperature of 320°C. Full scan range from m/z 350 to 1500 with resolution of 60000 (at m/z 200). The top 40 precursors of the highest abundant in the full scan were selected and fragmented by higher energy collisional dissociation and analyzed in MS/MS. The resulting spectra from each fraction were searched separately by the search engines: Proteome Discoverer 2.2 (PD 2.2, Thermo). Proteins containing similar peptides that could not be distinguished by MS/MS analysis were identified as a same protein group. The protein quantitation results were statistically analyzed by Mann-Whitney Test, for proteins whose quantitation significantly different between experimental and control groups. GSEA analysis was performed using desktop application (<http://www.broadinstitute.org/gsea/index.jsp>) based on GO database, which were acquired from the Molecular Signatures Database (MSigDB). Venn analysis was performed in the website (<http://bioinformatics.psb.ugent.be/webtools/Venn/>).

**Chromatin** **immunoprecipitation PCR**

ChIP was performed by using a ChIP assay Kit (Millipore, MA, USA) according to the manufacturer’s instructions and a ChIP antibody against H3K79me2 (#5427) purchased from Cell Signaling Technology (Danvers, MA, USA). The precipitated DNA fragments were quantified by real-time qPCR and normalized using the internal control IgG. The ChIP data was presented as a percentage relative to the input DNA amount by the equation: 2^[Input Ct – Target Ct]^ × 100. Primer sequences are listed in Supplemental table 7.

**Co-immunoprecipitation (Co-IP)**

Co-IP was done using the Thermo Scientific Pierce Co-IP kit (26149) following the manufacturer’s protocol. Briefly, the specific antibody was first immobilized for 2 hours using AminoLink Plus coupling resin. Cells were collected and lysed in applicable IP lysis buffer. The resin was then washed and incubated with cell lysate overnight. After incubation, the resin was again washed and protein was eluted by using elution buffer. A negative control was provided with IgG. Samples were analyzed by immunoblotting analysis.

**Wound-healing assay**

HPMCs were seeded in a 6-well plate and allowed to reach 90% conﬂuence. A scratch wound was created on the cell surface using a micropipette tip. Then, cells were washed with PBS in three times and incubated in serum-free DMEM/F12 with TGF-β1 (2 ng/ml) in the presence or absence of EPZ5676 (10 μM), or with high glucose (60mM) in the presence of scrambled siRNA or DOT1L siRNA. Photomicrographs (×40 objective magnification) of migrating cells were taken at 0, 18 and 36 hours. The width of the wound was measured using ImageJ software. The migratory rate was calculated as (A − B)/A × 100%, where A and B reﬂect the width of the wound at 0, 18 and 36 h respectively.

1. Shi Y, Tao M, Wang Y, Zang X, Ma X, Qiu A, et al. Genetic or pharmacologic blockade of enhancer of zeste homolog 2 inhibits the progression of peritoneal fibrosis. J Pathol*.* 2020;250(1):79-94.<https://doi.org/10.1002/path.5352>.

2. Shi Y, Li J, Chen H, Hu Y, Tang L, Wang Y, et al. Inhibition of EZH2 suppresses peritoneal angiogenesis by targeting a VEGFR2/ERK1/2/HIF-1α-dependent signaling pathway. J Pathol*.* 2022;258(2):164-178.<https://doi.org/10.1002/path.5987>.

3. Vatapalli R, Sagar V, Rodriguez Y, Zhao JC, Unno K, Pamarthy S, et al. Histone methyltransferase DOT1L coordinates AR and MYC stability in prostate cancer. Nat Commun*.* 2020;11(1):4153.<https://doi.org/10.1038/s41467-020-18013-7>.
